# EPA guidance on lifestyle interventions for adults with severe mental illness: A meta-review of the evidence

**DOI:** 10.1192/j.eurpsy.2024.1766

**Published:** 2024-12-10

**Authors:** Isabel Maurus, Sarah Wagner, Johanna Spaeth, Anastasia Vogel, Susanne Muenz, Valentina Seitz, Peter von Philipsborn, Marco Solmi, Joseph Firth, Brendon Stubbs, Davy Vancampfort, Mats Hallgren, Tamás Kurimay, Markus Gerber, Christoph U. Correll, Wolfgang Gaebel, Hans-Jürgen Möller, Andrea Schmitt, Alkomiet Hasan, Peter Falkai

**Affiliations:** 1 Department of Psychiatry and Psychotherapy, University Hospital, LMU Munich, Munich, Germany; 2 Institute for Medical Information Processing, Biometry and Epidemiology (IBE), Munich, Germany; 3 Pettenkofer School of Public Health, Ludwig-Maximilians-University Munich, Munich, Germany; 4 Department of Psychiatry, University of Ottawa, Ontario, Canada; 5 Regional Centre for the Treatment of Eating Disorders and On Track: The Champlain First Episode Psychosis Program, Department of Mental Health, The Ottawa Hospital, Ontario, Canada; 6 Ottawa Hospital Research Institute (OHRI) Clinical Epidemiology Program, University of Ottawa, Ontario, Canada; 7 Department of Child and Adolescent Psychiatry, Charité Universitätsmedizin Berlin, Berlin, Germany; 8 Division of Psychology and Mental Health, University of Manchester, Manchester Academic Health Science Centre, Manchester, UK; 9 Institute of Psychiatry, Psychology and Neuroscience, King’s College London, London, UK; 10 KU Leuven Department of Rehabilitation Sciences, Leuven, Belgium; 11 Department of Public Health Sciences, Karolinska Institutet, Solna, Sweden; 12 Institute for Physical Activity and Nutrition (IPAN), Deakin University, Melbourne, Australia; 13 North-Buda Saint John Central Hospital, Buda Family Centered Mental Health Centre, Department of Psychiatry and Psychiatric Rehabilitation, Teaching Department of Semmelweis University, Budapest, Hungary; 14 Department of Sport, Exercise and Health (DSBG), University of Basel, Switzerland; 15 Department of Psychiatry, The Zucker Hillside Hospital, Northwell Health, Glen Oaks, NY, USA; 16 Department of Psychiatry and Molecular Medicine, Donald and Barbara Zucker School of Medicine at Hofstra/Northwell, Hempstead, NY, USA; 17 German Center for Mental Health (DZPG), Berlin, Germany; 18 Department of Psychiatry and Psychotherapy, Medical Faculty, Heinrich-Heine University, Duesseldorf, Germany; 19 WHO Collaborating Centre DEU-131, LVR-Klinikum Düsseldorf, Duesseldorf, Germany; 20 Laboratory of Neuroscience (LIM27), Institute of Psychiatry, University of Sao Paulo, São Paulo, Brazil; 21 Department of Psychiatry, Psychotherapy and Psychosomatics, Faculty of Medicine, University of Augsburg, Bezirkskrankenhaus Augsburg, Augsburg, Germany; 22 German Center for Mental Health (DZPG), Munich/Augsburg, Germany; 23 Max Planck Institute of Psychiatry, Munich, Germany

**Keywords:** affective disorder, diet, exercise, schizophrenia, sleep

## Abstract

There is growing interest in lifestyle interventions as stand-alone and add-on therapies in mental health care due to their potential benefits for both physical and mental health outcomes. We evaluated lifestyle interventions focusing on physical activity, diet, and sleep in adults with severe mental illness (SMI) and the evidence for their effectiveness. To this end, we conducted a meta-review and searched major electronic databases for articles published prior to 09/2022 and updated our search in 03/2024. We identified 89 relevant systematic reviews and assessed their quality using the SIGN checklist. Based on the findings of our meta-review and on clinical expertise of the authors, we formulated seven recommendations. In brief, evidence supports the application of lifestyle interventions that combine behavioural change techniques, dietary modification, and physical activity to reduce weight and improve cardiovascular health parameters in adults with SMI. Furthermore, physical activity should be used as an adjunct treatment to improve mental health in adults with SMI, including psychotic symptoms and cognition in adults with schizophrenia or depressive symptoms in adults with major depression. To ameliorate sleep quality, cognitive behavioural informed interventions can be considered. Additionally, we provide an overview of key gaps in the current literature. Future studies should integrate both mental and physical health outcomes to reflect the multi-faceted benefits of lifestyle interventions. Moreover, our meta-review highlighted a relative dearth of evidence relating to interventions in adults with bipolar disorder and to nutritional and sleep interventions. Future research could help establish lifestyle interventions as a core component of mental health care.

## Introduction

For many illnesses, lifestyle interventions are seen as the cornerstone of prevention and first-line treatment, because they can improve key clinical outcomes and are generally associated with low risks for side effects [1–5].

At the same time, there is an urgent need to expand standard treatment options for people with severe mental illness (SMI). SMI, defined as schizophrenia spectrum disorders, bipolar and major depressive disorders in this work [6], lead to a high symptom burden, low levels of functioning in daily life and have very often unfavourable outcomes due to persistent psychiatric symptoms [7]. Many affected individuals do not benefit sufficiently from standard psychological and psychopharmacological approaches; in addition, several psychotropic medications are frequently accompanied by adverse side effects on many health aspects, such as weight-gain and metabolic syndrome [8, 9], resulting often in a lower treatment adherence [10]. SMI is also associated with significant somatic comorbidity. Life expectancy in patients with SMI is reduced by 10 to 20 years compared to the general population, with cardiopulmonary and cardiovascular diseases being among the leading causes of death [11–14]. Further, reduced access to healthcare and mental-health-related stigma in healthcare lead to lower rates of screening and treatment of cardiovascular disease in patients with SMI [15, 16]. Apart from increased rates of alcohol and tobacco consumption [17], unhealthy nutrition [18], sleep difficulties [19], physical inactivity, and sedentary behaviour [20, 21] are more prevalent in patients with SMI, and although modifiable, they contribute to the markedly higher prevalence of cardiovascular disease in this clinical population. For example, the diet of adults with SMI is on average less healthy and more obesogenic than the diet of the general population [22] and adults with SMI spend on average about 8 h per day being sedentary during waking hours, which is considerably more than observed in the general population [20]. Notably, physical inactivity [23], insomnia [24, 25], and obesity [26–28] are risk factors contributing not only to chronic cardiovascular diseases, but also to the development or aggravation of mental illnesses. While schizophrenia is typically associated with the greatest degree of sedentary behaviour, unhealthy diet and cardiovascular risk [20, 22], there is compelling evidence that also people with major depression and bipolar disorder have an increased risk of obesity, diabetes and metabolic syndrome that is at least twice as high as in the general population [29–31]. Consequently, the 2018 World Health Organization (WHO) guidelines on the management of physical health conditions in adults with SMI recommend lifestyle interventions as first-line strategies for the prevention of cardiovascular diseases in this population [32]. Lifestyle interventions usually show high acceptability among patients [33] and are increasingly recognized as fundamental for both physical and mental health.

To date, physical activity, including exercise interventions, is the most widely researched lifestyle behaviour in people with SMI and has the potential to improve psychiatric symptoms, cognition, and levels of functioning across a range of mental health diagnoses [34]. These findings were synthesised in a guidance paper by Stubbs et al. published in 2018 [35]. It concluded that physical activity improves depressive symptoms, quality of life and cardiorespiratory fitness in major depressive disorder, and psychiatric symptoms and cognition in schizophrenia spectrum disorders. The current meta-review aims to update this guidance and extend it, considering the efficacy of physical activity interventions when combined with other lifestyle interventions and other lifestyle interventions alone. In general, in the exercise (i.e., structured physical activity) research field, progress is often hampered by inconsistent and imprecise intervention definitions and descriptions, implemented in different target populations and settings with wide variations in the type, frequency, intensity, and duration of interventions in many studies [36, 37]. This problem is even more pronounced in other, less researched lifestyle domains [38, 39], which makes it difficult to draw consistent conclusions about the efficacy and effectiveness of interventions and to formulate specific recommendations about their use.

Accordingly, in this guidance paper, we sought to identify the top-tier evidence on lifestyle interventions for adults with SMI to assist in expanding the range of options available to clinicians. This guidance paper consists of two components. The first component is a meta-review of systematic reviews on the effects of non-pharmacological interventions to improve physical activity, diet, and sleep in adults with SMI. We thereby aimed to summarize both their impact on modifiable risk factors for somatic disorders and physical health and to provide new insights about their benefits on mental health outcomes and quality of life. The second component involves using the synthesis of the data to develop a set of practice recommendations and research priorities using the guidance framework of the European Psychiatric Association (EPA). Our goal is to synthesize the existing literature and to mobilize research knowledge into an actionable implementation plan to guide service delivery and to address key research gaps.

## Methods

### Guidance development process

This meta-review (PROSPERO registration ID CRD42022307336) followed PRISMA guidelines [40] implemented through a pre-determined, published protocol compiled by a research team with different and complementary research expertise within lifestyle-based interventions for mental health. Our team comprises several professional groups, including physicians, psychologists, physiotherapists, sports and exercise scientists, and public health researchers affiliated with institutions from different European countries. The guidance for clinical practice and research was conducted in accordance with the EPA guidelines framework [41].

### Definition of severe mental illness

In the literature, different definitions are used to describe SMI. In the present project, we used the definition of the U. S. National Institute of Mental Health (NIMH) of SMI as “a mental, behavioural, or emotional disorder resulting in serious functional impairment, which substantially interferes with or limits one or more major life activities” [6], and is focused on major depression, bipolar disorder and schizophrenia spectrum disorders.

### Definition of lifestyle interventions

Currently, there is no consensus definition of the term “lifestyle intervention” which comprises a wide range of interventions. In the medical context, lifestyle interventions usually comprise physical activity, diet, adequate sleep and stress management [42]. However, while physical activity, diet and sleep are considered ‘typical’ health behaviours, stress management techniques are a relatively new area of interest where interventions are even less clearly operationalized [43]. To ensure the feasibility and applicability of this EPA guidance paper, we focused on a narrower definition of lifestyle interventions and restricted the content to non-pharmacological lifestyle interventions focused on physical activity, diet, or sleep interventions. In this meta-review, we use the term physical activity in a broad sense to include interventions to reduce sedentary behaviour and increase structured physical activity, also referred to as exercise or sports interventions. We excluded interventions relating to alcohol or tobacco use, which are often considered psychiatric co-morbidities and have been covered in previous EPA guidance papers and other guidelines on the management of substance use disorders [44, 45]. We included mono- and multimodal interventions, which could have included educational or cognitive-behavioural components, or both, delivered in any setting and in any format.

### Systematic search and study identification

Two authors independently searched the databases PubMed, Cochrane Database of Systematic Reviews and Epistemonikos from inception to 16^th^ September 2022 and the database EMBASE from inception to 7 October 2022 for systematic reviews of primary studies investigating lifestyle interventions in adults with SMI. A subsequent updated search for new literature was conducted on 11 March 2024. Since an EPA guidance paper on physical activity for the population of SMI was published in 2018 [35] using overlapping search criteria, the search in the domain of physical activity focused on literature published after the period already covered (i.e., after 16 January 2018). The search was organized in accordance with the population, interventions, comparisons, outcomes, and setting/study (PICOS) reporting structure. Table 1 provides an overview of the search terms included in the overall search strategy, whereas Supplementary material S1 contains the terms for the detailed search strategies adapted to the syntax of different databases. In addition, the reference lists of the included articles were hand-searched to identify further potentially relevant reviews.

**Table 1.** Application of the PICOS search strategy for the main search terms included in the overall search strategy

**Population**
Severe mental illness; serious mental illness; severe mental disorder; serious mental disorder; major depressive disorder; schizophrenia spectrum and other psychotic disorders; bipolar disorder
**Interventions**
Health behaviour; lifestyle intervention; lifestyle medicine; lifestyle psychiatry; weight loss; weight reduction; weight management; diet; diet therapy; nutrition; exercise; physical activity; sport; yoga; sleep; sleep hygiene; sleep quality; improved sleep; sleep intervention
**Comparisons**
Not defined
**Outcomes**
Not defined
**Setting**
Delivered in any setting and in any format
**Study design**
Guideline; systematic review; meta-analysis
**Additional filters**
**Time period**
From inception, except for physical activity-related search terms (published after 16th January 2018)
**Language**
English
**Optional filter**
Humans

### Type of studies eligible for inclusion

The inclusion criteria were

- systematic reviews with or without meta-analyses that assessed studies of any design;
- listed in the databases PubMed, Cochrane Database for Systematic Reviews, Epistemonikos or EMBASE and published in English;
- that included adults with schizophrenia spectrum and other psychotic disorders, major depressive disorder or bipolar disorder (with or without cardiovascular comorbidity);
- that assessed non-pharmacological lifestyle interventions related to the treatment of mental disorders, targeting physical activity, weight, diet or sleep, including educational or cognitive-behavioural informed interventions, or both, delivered in any setting and in any format;
- that included any control;
- that considered outcomes regarding targeted health behaviours (e.g., amount of physical activity or reduction of sedentary behaviour), cardiovascular risk factors, psychopathology measures or assessments of the patients’ quality of life or functional outcomes.

We excluded

- conference abstracts and studies that were meta-reviews or guidelines that did not employ a systematic search;
- systematic reviews of studies in which <50% of participants in the intervention groups had any of the mental disorders under review;
- reviews that included people aged younger than 18 years;
- reviews that included people that were suffering from disorders other than those defined as SMI here (e.g. seasonal affective disorders, premenstrual dysphoric disorder, peri- and post-partum depression/psychosis) or patients in palliative care;
- reviews that assessed pharmacological interventions, stress management (e.g., meditation or mindfulness), dietary interventions solely focused on the intake of specific preparations (e.g., supplements, vitamins) or interventions involving the passive use of objects such as bright light lamps or blue-blocking glasses;
- reviews that focused on mechanistic factors of psychopathology as outcomes (e.g. brain structural changes).
- reviews considering other disorders or interventions in addition to the specified ones were excluded if the results of the subsets were not reported separately.
  Further details on inclusion and exclusion criteria are presented in the Supplementary material S2.

### Selection of articles and data extraction

A pair of authors (IM + AS or JS + SM) screened the identified articles on the title and abstract level independently for possible inclusion. Following the initial screen, the full text of potentially eligible reviews was assessed by two authors independently (AS+JS or SM + SW) for eligibility and disagreements were resolved through discussion among all authors (IM, AS, JS, SM, SW). If necessary, review authors were contacted for additional information. The same procedure was applied to potentially eligible articles identified in the reference lists of retrieved articles. Subsequently, we extracted the following data from eligible full-text articles for synthesis: Type of review, search span, study designs included in the review, number of studies included, sample size of the population of interest, mean age of the population, details of the interventions, control groups, outcome measures, main results and conflicts of interest reported.

### Quality assessment included systematic reviews and grading of evidence

To assess the certainty of the evidence, we evaluated the quality of the included systematic reviews and meta-analyses using the SIGN Methodology Checklist for Systematic Reviews and Meta-analyses [46] adapted from the AMSTAR tool [47], as indicated by the EPA guidelines [48]. Accordingly, the quality of each review was rated as high (++), acceptable (+), low (−) or unacceptable (0) (see Table 2). Discrepancies in the ratings were resolved by discussion (AS, JS, SM, SW, IM).

**Table 2.** Overall assessment of the methodological quality of reviews according to the SIGN Methodology Checklist for Systematic Reviews and Meta-analyses

**High quality (++)**
Majority of criteria met. Little or no risk of bias.
**Acceptable (+)**
Most criteria met. Some flaws in the study with an associated risk of bias.
**Low quality (−)**
Either most criteria not met, or significant flaws relating to key aspects of study design.
**Unacceptable - reject (0)**
Poor quality study with significant flaws. Wrong study type. Not relevant to guideline.

### Guidance development

To develop recommendations, we considered the evidence identified in our systematic review and the quality of reviews as assessed by the SIGN Methodology Checklist [46]. Recommendations were then graded according to the EPA guidance framework (see Table 3) and reviewed by all the authors.

**Table 3.** Grading of recommendations

Grade	
A	At least one review rated as High quality and directly applicable to the target population, OR a body of evidence consisting principally of reviews rated as High quality, directly applicable to the target population, and demonstrating overall consistency of results
B	A body of evidence including reviews rated as Acceptable, directly applicable to the target population, and demonstrating overall consistency of results OR extrapolated evidence from reviews rated as High quality or Acceptable
C	A body of evidence including reviews rated as acceptable to low quality, directly applicable to the target population, and demonstrating overall consistency of results or extrapolated evidence from reviews rated as acceptable or low quality.
D	Reviews rated as Low quality or Unacceptable OR extrapolated evidence from reviews rated as Low quality, Unacceptable OR expert consensus.

Duplicates removed (n = 584)Papers excluded based on title/abstract(n = 2614)Additional records identified through hand searching and screening of lists of references (n = 16)Duplicates removed (n = 584)Papers excluded based on title/abstract(n = 2614)Additional records identified through hand searching and screening of lists of references (n = 16).

*Modified from Gaebel W, Großimlinghaus I, Mucic D, Maercker A, Zielasek J, Kerst A. EPA guidance on eMental health interventions in the treatment of posttraumatic stress disorder (PTSD). Eur Psychiatry. 2017;41(1):140–52.*

## Results

**Figure 1.**
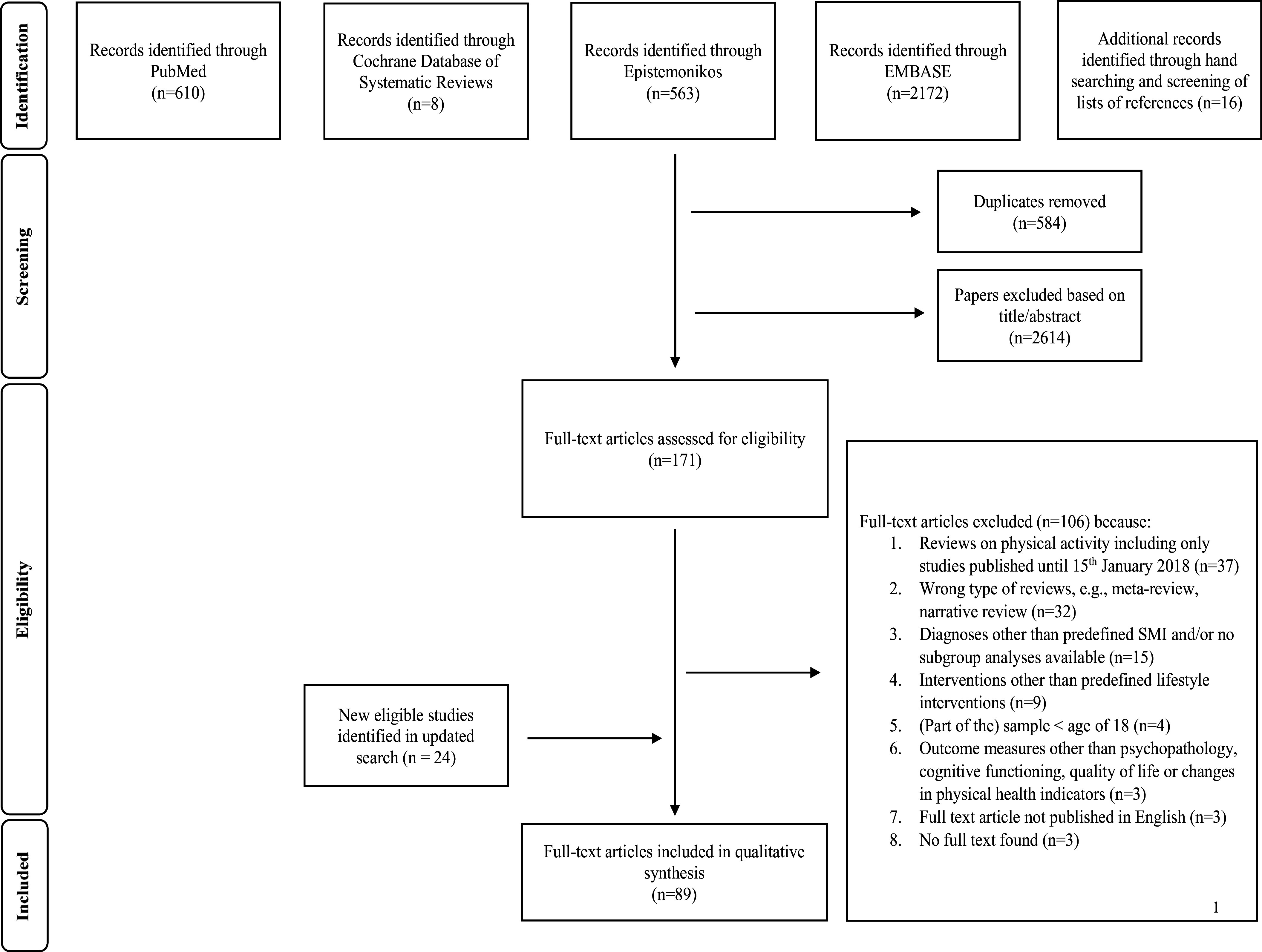
PRISMA flowchart (from Page et al. [40]).

The initial database searches yielded 3353 records and 16 additional records from hand search. After removing duplicates, 2785 records were reviewed at the title and abstract level, of which 2614 were excluded. Subsequently, we assessed 171 full-text articles, of which 106 were excluded (see Figure 1 below). The subsequent updated search for new literature resulted in a further 24 included full-texts. Overall, 89 systematic reviews and meta-analyses were included in this guidance paper. The details of the systematic search process are shown in Figure 1. A list of the excluded full-text reviews of the primary search is given in Supplementary material S3, while a summary of the included reviews is presented in Supplementary materials S4–S7. The number of reviews included in this meta-review is given in Table 4.

**Table 4.** Number of reviews included in this meta-review

Type of interventions	SMI (65 reviews)			
	Mixed SMI	Major depressive disorder	Bipolar disorder	Schizophrenia spectrum disorders
	Number of reviews			
Multimodal	25	–	3	15
Physical activity	7	9	–	20
Diet	3	3	1	1
Sleep	–	–	1	1

SMI = severe mental illness.

### Multimodal lifestyle interventions

Most of the reviews identified cover different types of lifestyle interventions. As primary studies often used combined interventions whose effects were reported jointly, their findings are reported in a separate category on multimodal lifestyle interventions. Details on the included reviews can be found below and in Supplementary material S4.

#### Severe mental illnesses

We identified a total of 25 systematic reviews (including two updates from previous reviews) focusing on combined lifestyle interventions for mixed populations with SMI. Most of the reviews considered randomized controlled trials (RCTs), while some included also other study types such as quasi-experimental studies.

The interventions of interest consisted of various combinations of physical activity programs [39, 49–61], dietary changes including nutrition education and counselling [38, 39, 50–65], psychoeducational, motivational or cognitive behavioural techniques to modify behaviour towards healthier lifestyle choices [38, 49, 51, 53–56, 60–62, 66–68], peer or family support [49, 69], social media interventions aiming at increased physical activity or a healthier eating [49, 53, 54, 62], or the use of digital technologies targeting lifestyle behaviour or weight loss, such as mobile phone applications or pedometers [49, 62, 64].

In summary, interventions combining behaviour change techniques, dietary modification and physical activity were found to be effective in improving weight or body mass index (BMI) in adults with SMI [49–51, 53, 55, 56, 58, 59, 65–67]. However, the clinical relevance of findings was criticised repeatedly in the systematic reviews (usually only a weight loss of more than 5% is considered as clinically relevant to reduce health-related complications) [53, 56, 67], and more consistent results were found, especially for interventions with a study duration of more than 6 months [52, 57]. Several effective elements of these behavioural interventions were identified, including regular contact, supporting tools, such as intervention handbooks or pedometers, and educational materials [59]. In addition, multimodal interventions were shown to decrease distinct aspects of the metabolic syndrome, such as fasting glucose levels [50, 51, 57] or triglyceride levels [50, 51].

Interventions using digital technologies (such as pedometers or mobile phone communication) have been reported to be easy to use and to reduce sedentary behaviour [64]. Social media interventions were also considered highly acceptable, but only resulted in non-significant improvements in cardiorespiratory fitness [62]. The evidence on the effects of peer or family support remains inconclusive [69]. Most reviews focused on improvements in physical health, and mental health outcomes were less consistently reported. Reviews that examined effects on quality of life showed heterogeneous results [38, 54, 56, 58, 61, 67, 68], whereas effects on depressive symptoms [50, 54] or anxiety [54] were more consistently detected. In general, delivering interventions both in a group setting as well as in individual sessions has been recommended [38, 56]. The quality of the included reviews was variable, ranging from unacceptable to high [54, 56, 57, 59, 61, 62, 68].

##### Recommendation (Grade A)

Lifestyle interventions that combine behaviour change techniques, dietary modifications, and physical activity should be offered to individuals with SMI for reducing weight and other metabolic syndrome components, as well as depressive and anxiety symptoms.

#### Major depressive disorder

We did not find any systematic reviews that separately investigated the effects of mixed lifestyle interventions in adults with major depressive disorder.

#### Bipolar disorder

Three systematic reviews reporting data from 1898 participants considered individuals with bipolar disorder [70–72]. The reviews included RCTs [72] as well as non-randomized controlled studies [70, 71]. Two reviews focused on multimodal lifestyle intervention programs covering domains such as dietary modification, physical activity, sleep, and motivational or self-management aspects, but identified few primary studies that met the inclusion criteria [70, 72]. In the systematic review by Bauer et al., weight or BMI was the primary outcome in most of the trials. Overall, results pointed towards beneficial effects on weight, blood pressure, lipid profile, physical activity and mood, but the quality of the underlying studies was criticized due to small sample sizes, variability in assessments and an insufficient number of RCTs [70]. The quality of the review was rated as acceptable according to SIGN. The systematic review by Simjanoski et al. concluded that combined interventions covering diet, physical activity and sleep reduced depressive symptoms and improved overall functioning [72]. The quality of this review was rated as high.

Another review examined the effects of e-Health (health services and information via the internet) and m-Health (use of mobile devices) interventions to improve self-management, which included maintaining a healthy lifestyle as well as topics such as mood or medication monitoring, or self-education about the illness [71]. However, only few primary studies incorporated lifestyle aspects. Review quality was rated as low.

##### Recommendation (Grade B)

Multimodal lifestyle interventions can be used to improve overall functioning and reduce depressive symptoms as well as cardiovascular risk factors in adults with bipolar disorder.

#### Schizophrenia spectrum disorders

A total of 15 systematic reviews summarized the effects of multimodal lifestyle interventions in adults with schizophrenia spectrum disorders. Most reviews included RCTs [73–79], while some also included studies with non-randomized designs, such as quasi-experimental trials, case studies, cohort or retrospective studies [80–82].

The interventions of interest mostly involved weight management programs that incorporated nutritional education and counselling or caloric restriction [73–75, 77, 80–87], physical activity promotion or exercise programs [73–78, 80–82, 84, 86], and cognitive behavioural or motivational elements (such as social reinforcement, token economy, goal setting, progress monitoring) [73–77, 81, 84, 87].

All the reviews assessed the effects of lifestyle interventions on body weight or BMI as the main outcome, and all found small to moderate improvements. Other cardiometabolic risk factors, such as waist circumference [75, 76], blood glucose levels [82] or cardiorespiratory fitness [84] were also improved. Peer support was found to increase the amount of physical activity in daily life and to improve cardiorespiratory fitness [79].

Mental health outcomes were less commonly reported. Effects on quality of life were inconsistent [73, 75, 78], while beneficial effects on positive, negative or general symptoms and cognition were shown particularly in programs incorporating physical activity elements [75, 76]. The quality of the included reviews ranged from unacceptable to high quality [73–78, 82].

These findings collectively suggest that programs combining both physical activity, nutritional counselling and motivational or behavioural change techniques may be the most successful.

##### Recommendation (Grade A)

To improve healthy weight management in individuals with schizophrenia spectrum disorders, multicomponent programs that include physical activity, nutritional counselling and motivational and/or cognitive behavioural techniques should be offered.

### Physical activity

In total, we identified 36 systematic reviews dealing with the effects of physical activity as the sole intervention. Details on the included reviews are given below and in Supplementary material S5.

#### Severe mental illnesses

In our systematic search to complement the recent EPA guidance paper on physical activity in SMI [35], we found seven new systematic reviews that included mixed SMI populations [88–94], five of which conducted a meta-analysis.

Three reviews included only RCTs [90, 92, 94], while the remainder also considered non-randomized controlled trials as well as pre-post intervention studies [88, 89, 91, 93]. Four reviews studied a variety of physical activity interventions [88, 91, 92, 94]. Others addressed specific types of exercise, including yoga [90] and high-intensity interval training (HIIT) [89] or digital interventions [93]. One review focused only on inpatients [91]. To date, it is unclear whether physical activity interventions are effective in improving physical health parameters [91] or in increasing general physical activity levels in the daily lives of individuals with an SMI [88]. Similarly, digital health behaviour interventions showed mixed results [93]. With respect to depressive symptoms, yoga appears to be more effective than various inactive control conditions such as educative sessions, waitlists or TAU [90]. High-intensity interval training may also be a highly acceptable and safe form of exercise with potential antidepressant effects [89]. Another systematic review concluded that especially multimodal exercise interventions are beneficial in relieving depressive symptoms [94].

Moreover, physical activity has been shown to have positive effects on several cognitive domains, such as reasoning and problem solving [92]. Physical activity interventions that aim to achieve at least a moderately intense level of physical exertion [95] and a minimum of 150 min per week can generally be recommended to improve the mental health condition in individuals with SMI [5].

The quality of individual reviews according to SIGN ranged from unacceptable to high quality [91, 92, 94]. The recommendation from the previous EPA guidance paper on physical activity [35] can be updated as follows:

##### Recommendation (Grade A)

Physical activity should be offered to individuals with SMI for improving depressive symptoms and cognitive functioning.

#### Major depressive disorder

Nine recent reviews, each supplemented with a meta-analysis, were included focusing on major depressive disorder [96–104]. The primary studies included in the reviews were almost exclusively RCTs of physical exercise in general [97], aerobic exercise [104], or aerobic and strength training with [96] or without [101, 103] meditative movements. One review investigated yoga interventions only [102], one reviewed qigong-based therapy [98], while another distinguished between exercise, yoga, and tai chi [99]. Despite some null findings [103], aerobic and resistance exercise appears to be superior in improving depressive symptoms [99–101] when compared to various control conditions [97]. Moreover, aerobic exercise interventions showed beneficial effects on overall cognitive functioning and the subdomains of memory and executive function [104]. In addition, different types of physical activity may improve sleep quality, with mind–body exercise or vigorous strength training [96] showing the most promising results. Add-on yoga may also improve depressive symptoms [102], although the quality of evidence was low to moderate [102], and the effect was reduced when only studies with a low risk of bias were considered [99]. To date, evidence for an antidepressant effect of other mind–body exercise forms, such as tai chi [99] and qigong [98], is insufficient. Further, no conclusions can be drawn about the physical health benefits and effects on the quality of life [103] of physical activity in individuals with major depressive disorder. Except for two reviews, we rated the quality of reviews as acceptable to high [96, 99, 101–104]. Accordingly, the updated recommendation is as follows:

##### Recommendation (Grade A)

Physical activity should be offered as a treatment to improve depressive symptoms, cognitive functioning, and sleep quality in adults with major depressive disorder. Among meditative forms of physical activity, the strength of evidence is greatest for yoga.

#### Bipolar disorder

As with the previous guidance paper on physical activity [35], no systematic reviews could be identified focusing on the effects of physical activity in adults with bipolar disorder. To fill this research gap, future work should aim to synthesise the existing findings in this population.

#### Schizophrenia spectrum disorders

Twenty recent systematic reviews reported on the effects of physical activity in individuals with schizophrenia spectrum disorders, 16 of which included a meta-analysis [37, 95, 105–118]. Twelve reviews analysed only RCTs [37, 95, 107, 108, 110, 112, 114, 115, 117–120], and the remainder also included non-randomized controlled trials [105, 106, 109, 111, 113, 116, 121, 122].

Interventions were defined as any form of physical activity [37, 95, 106, 110, 113, 115, 116, 118, 120, 122], aerobic or resistance exercise [37, 105, 107, 109, 111], and mind–body exercises such as yoga and tai chi [107, 108, 112, 114, 117]. Two reviews focused on the mode of intervention delivery, including group-based [121] and videogame-based exercise [119].

Physical activity interventions in general were shown to be effective in alleviating positive [115, 116], general [116], and especially negative symptoms of schizophrenia spectrum disorders [37, 95, 107, 111, 113, 116, 118, 120] as well as the quality of life [95]. Moreover, improvements in cognitive functioning and functioning in daily life have been found [120]. Effects on depressive symptoms were mixed [95, 115]. While the effects of resistance exercise or exercise combinations, including resistance exercise on PANSS scores were limited by the small number of studies [107, 111], there is particularly strong evidence for aerobic exercise [37, 107, 110, 111]. Further, aerobic exercise can reduce PANSS positive and general symptoms [110, 111] and improve cognitive functioning [105, 109]. Enhancements in global cognition and several cognitive subdomains appear to be particularly supported for group-based [105, 121] and supervised [105, 109] aerobic interventions. Overall, benefits were found regardless of the type of exercise performed [113], including interventions with mind–body exercise components [95] and multicomponent interventions [115].

Mind–body exercise, such as yoga and tai chi, may also be more effective in reducing negative symptoms than various control conditions [107, 112, 114, 117]. However, there is currently a lack of specific evidence for tai chi [112]. With respect to yoga interventions, three reviews reported beneficial effects of yoga compared to treatment as usual on positive [108, 117] or negative symptoms [114, 117]. However, another review found no significant differences [110], while a fourth reported that positive effects of yoga on these symptoms were no longer significant after excluding studies with a high risk of bias or outliers [112]. In general, reported effects of interventions including mind–body exercises were more heterogeneous than those without, potentially indicating higher instructor-dependence [95].

No consistent effects of physical activity interventions on body weight could be detected [95, 118].

There is still insufficient research on the optimal delivery of physical activity interventions. While there is only very limited evidence for videogame-based training [119], we found the most support for professionally supervised group exercise [95, 105, 121]. In addition, higher levels of physical activity generally appear to be associated with increasing benefits for positive, negative and general symptoms [95, 106], but different recommendations have been made in terms of the minimum duration and intensity required [37, 105]. While some reviews suggested shorter durations [105, 118], as general agreement at least moderate-intensity exercise [37, 95] should be achieved in sessions lasting more than 120 min per week [37, 118] (150 min per week according to the WHO [5]) for more than 6 months [37, 115]. In general, more frequent sessions seem to be more effective in improving psychosocial functioning [95].

Albeit the quality of the primary research was repeatedly criticized, the reviews were almost exclusively of high quality. Therefore, the recommendation from the previous EPA guidance paper [35] can be updated as follows:

##### Recommendation (Grade A)

Physical activity should be offered to individuals with schizophrenia spectrum disorders as an adjunctive treatment to improve positive, negative and general psychopathology symptoms, cognition, and quality of life.

### Diet

Eight different systematic reviews were found investigating dietary interventions. Details on the included reviews are given below and in Supplementary material S6.

#### Severe mental illnesses

Three of the eight systematic reviews focused on dietary interventions for individuals with SMI [123–125]. Due to the combined reporting of results from clinical populations with depression and anxiety, we could consider only one study (*n* = 16) conducted in individuals with schizophrenia from the systematic review by Aranburu et al. [123], which examined the effects of a gluten-free versus gluten-containing diet. The dietary interventions reported by Teasdale et al. consisted of individualized counselling, group education, or a combination of these [124]. Significant reductions in weight, BMI, and waist circumference were reported compared to treatment as usual, leading the authors to conclude that dietary interventions may be beneficial for weight control in SMI, particularly when delivered by dietitians [124]. The systematic review by Rocks et al. also emphasises the importance of interventions delivered by dietitians and in individual sessions, but found only limited evidence for the effectiveness of dietary interventions in improving metabolic syndrome risk factors in people with SMI in general [125]. The quality of this review was rated as high [125], while the other two reviews were rated as low or unacceptable. Due to the limited amount and quality of the available evidence, no firm conclusions can currently be drawn from these reviews for adults with SMI.

#### Major depressive disorder

We identified three systematic reviews that investigated the impact of dietary interventions on depressive symptoms. The interventions included a 6-month Mediterranean diet [126], different forms of caloric or carbohydrate restriction [127], or Intermittent (Ramadan) Fasting [128]. Intermittent Fasting had a beneficial effect on anthropometric outcomes, but the review quality was rated as low. Both the Mediterranean diet or caloric restriction interventions appeared to be associated with fewer depressive episodes [126] or mood improvements [127] when compared with the control conditions. However, only one review was of acceptable quality [127], and there was considerable heterogeneity between studies, which limits the generalisability of the results. Further interventional research is therefore needed to formulate more robust dietary recommendations for patients with major depressive disorder.

##### Recommendation (Grade D)

Dietary interventions may be considered to improve depressive symptoms in adults with major depressive disorder.

#### Bipolar disorder

One systematic review aimed to examine the effectiveness of obesity interventions in adults with bipolar disorder, but found no studies that met the inclusion criteria, highlighting the need for research in this area [129].

#### Schizophrenia spectrum disorders

Only one systematic review was identified that aimed to investigate dietary interventions as a single lifestyle modification in adults with schizophrenia [130]. However, the authors did not find any studies that met the inclusion criteria [130]. Therefore, currently, no recommendation can be made regarding dietary interventions alone in adults with schizophrenia spectrum disorders. Future experimental studies are needed to identify the most beneficial dietary patterns and food components in this population.

### Sleep interventions

We found two systematic reviews that investigated sleep interventions. Details on these reviews are provided below and in the Supplementary material S7.

#### Severe mental illnesses

There were no systematic reviews that summarised the results of sleep interventions in mixed SMI populations.

#### Major depressive disorder

We did not find any systematic reviews of sleep interventions for adults with major depressive disorder.

#### Bipolar disorder

One review investigated non-pharmacological interventions targeting sleep and circadian rhythms in adults with bipolar disorder and included 10 primary RCTs [131]. Interventions were heterogeneous, ranging from sleep deprivation (*n* = 1), interpersonal and social rhythm therapy to improve sleep behaviour (*n* = 4), cognitive behavioural therapy-informed intervention for insomnia (*n* = 1) and combination treatments (*n* = 4). In sum, interpersonal and social rhythm therapy to improve sleep behaviour yielded conflicting results on mood symptoms, while total sleep deprivation and combination therapies showed no effect. The only available study on cognitive behavioural therapy-informed intervention for insomnia found significant reductions in depression scores, improvements in insomnia symptoms and improved sleep quality. Of note, only a minority of primary studies included sleep or circadian rhythm as an outcome, focusing instead on affective symptoms [131]. Although the review was rated as high quality according to SIGN criteria, it provides only limited evidence for the effectiveness of the interventions in question due to the small number of studies per intervention, as well as the heterogeneity of intervention protocols, outcomes and patient characteristics. Thus, the current evidence does not allow firm conclusions to be drawn about the effects of sleep interventions in adults with bipolar disorder.

#### Schizophrenia spectrum disorders

We identified one systematic review [132] investigating the effectiveness of psychological interventions for sleep disturbances in adults with schizophrenia, of which a subset of four primary studies with a total of 263 participants was relevant to the present work. The review included RCTs and non-randomized trials with treatment as usual control groups and focused on cognitive behavioural therapy-informed interventions targeting insomnia or, in one case, nightmares. Treatment duration ranged from two to 12 weeks. The interventions led to sleep improvements, with large effect sizes post-treatment and at follow-up. The results also indicated modest improvements in positive symptoms, but the trials were not adequately powered to detect this effect in patient populations. Moreover, the quality of this review was rated as unacceptable according to SIGN criteria because its literature search was limited to one electronic database; therefore, no clear recommendations for the use of sleep interventions in adults with schizophrenia spectrum disorders can be made at this time based on the current evidence. More clinical trials, which should include outcomes of sleep and circadian rhythm in addition to psychopathological outcomes, are needed to determine the most beneficial intervention protocols.

##### Recommendation (Grade D)

Cognitive behavioural therapy-informed interventions targeting insomnia may be considered to improve sleep quality and mental health outcomes in adults with SMI.

A summary of all recommendation grades is given in Table 5.

**Table 5.** Summary of recommendation grades

Type of interventions	Mixed SMI	Major depressive disorder	Bipolar disorder	Schizophrenia spectrum disorders
	Grade of Recommendation			
Multimodal	A	–	C	A
Physical activity	A	A	–	A
Diet	–	D	–	–
Sleep	–	–	–	D

SMI = severe mental illness. Definitions of the recommendation grades are given in Table 3. A minus indicates a lack of systematic reviews (or their insufficient quality) to formulate a recommendation.

## Discussion

In this EPA Guidance paper, we conducted a meta-review to synthesise the evidence on lifestyle interventions for adults with SMI and formulate recommendations for both clinical practice and research in this area.

Many of the identified reviews included the mixed diagnosis group of SMI. In summary, we found that lifestyle interventions that combine behavioural change techniques, dietary modification and physical activity show the greatest promise in reducing weight and improving additional cardiovascular health parameters. Furthermore, in line with the previous research [34], we conclude that add-on physical activity can be used to improve also depressive symptoms in adults with SMI. Even if there is still insufficient evidence for the optimal delivery of exercise interventions, group-based sessions supervised by qualified professionals [133, 134] of at least moderate intensity [37, 95] for a total of more than 150 min per week [5] seem to be advantageous.

In adults with major depressive disorder, physical activity was shown to ameliorate depressive symptoms and improve cognitive functioning, sleep quality and physical fitness. A recent umbrella review also found significant effects of exercise interventions on physical health outcomes in this population [135], and associations between dietary interventions and reduced depressive symptoms have also been reported [126, 127]. In general, as noted before [35], there is still a lack of reviews on the benefits of lifestyle interventions for individuals with bipolar disorder. Nevertheless, multimodal lifestyle interventions can be a promising approach, offering potential benefits for improved cardiovascular risk factors, overall functioning and reduced depressive symptoms in people with bipolar disorder.

Most meta-research focused on lifestyle interventions in adults with schizophrenia spectrum disorders. Effective weight loss programs are multicomponent and incorporate physical activity, nutritional counselling, and motivational and/or cognitive behavioural techniques. In terms of mental health outcomes, physical activity is a useful adjunct treatment to improve schizophrenia-related positive, negative and general psychopathology, cognition and quality of life, which is in line with the previous literature [34]. Moreover, cognitive behavioural therapy-informed interventions can be considered to ameliorate sleep quality in adults with schizophrenia.

Our recommendations underscore the importance of lifestyle interventions in improving the physical and mental health and overall well-being of individuals with SMI. The strength of evidence varies across interventions and diagnoses, highlighting the importance of individualized approaches and further research.

We suggest that the following gaps in effectiveness and implementation research should be addressed in the future to further advance the field:

First, we recommend that future primary studies and reviews should examine and report the impact of lifestyle interventions on both mental and physical health outcomes such as symptom ratings, cognitive and social functioning, cardiovascular risk factors and also quality of life. One of the major strengths of lifestyle interventions is their ability to address several domains, and understanding the holistic benefits of these interventions is crucial. To date, most systematic reviews have focused on body weight as primary outcome. Lifestyle interventions can be beneficial even if they do not result in weight loss, and overemphasising body weight as outcome can demotivate the individuals affected and potentially increase (self-)stigmatisation [136]. Negative attitudes towards individuals with obesity are widespread also among healthcare professionals. Thus, it is crucial to ensure that research does not contribute to the perpetuation of stigma and self-stigma [136–140].

Second, our meta-review highlighted a relative dearth of evidence relating to interventions in adults with bipolar disorder. Therefore, future research should aim to fill the knowledge gaps in this population.

Third, we noted that physical activity is the best-investigated lifestyle intervention in people with SMI to date. Indeed, even within the latest WHO guidelines on physical activity and sedentary behaviour, schizophrenia and major depression are included among the conditions with sufficient evidence to recommend physical activity in the treatment [141]. Still, we need to improve our knowledge of the optimal delivery of physical activity interventions. Since there is no clear evidence that one type of exercise is superior to another, but that a minimum of 150 min of at least moderate-intensity physical activity per week is advisable [5], it is crucial to gain further insights on how interventions can be tailored to meet the individual needs of people with SMI and how people with SMI can be optimally supported to exercise regularly. To this end, contextual and motivational factors, such as social support [142], exercise history and preferences [143, 144], as well as exercise opportunities, including financial support [143], appear to be important and should be considered in exercise referrals [145–147].

Fourth, as previously noted [34], there is a current paucity of trials and reviews on the effects of sleep and dietary interventions and to date, mental illnesses have not been a specific focus within related public health guidelines. Therefore, future high-quality trials are needed to assess the therapeutic potential of these two important areas.

Fifth, clinicians currently lack appropriate tools to comprehensively assess lifestyle factors in standard care. Relying solely on anthropometric measures alone may result in delayed interventions, which could hinder the effective prevention of secondary diseases [134]. Behaviours related to diet (dietary patterns), physical activity (accelerometric data or self-report assessment tools, e.g., [148–150]), and sleep (sleep–wake cycle and quality assessments) should be considered alongside physical and metabolic assessments for a more accurate health risk evaluation.

Sixth, we need to gain a deeper understanding of the underlying biological, psychological, and social mechanisms through which lifestyle interventions impact mental health [34]. This will enable us to tailor lifestyle interventions to the unique needs of individuals with different SMI and disease phases (and with mental and physical multimorbidity) and to identify specific strategies and approaches that are most effective for different subgroups. Furthermore, we need to understand potential interactions between psychotropic medications, psychotherapy, and lifestyle interventions and how best to use lifestyle interventions in conjunction with established therapies.

Seventh, research should also focus on effective ways to implement lifestyle interventions within mental health treatment settings. This includes integrating these interventions into existing mental health care systems, training mental health professionals in their use, involving experts such as exercise physiologists, physiotherapists or nutritionists in their delivery, and understanding barriers and facilitators to implementation [134]. As lifestyle interventions represent a multifaceted treatment approach, we believe it is valuable to build interdisciplinary networks for delivery and to regularly seek input from allied health professionals [134]. In addition, technology-based interventions, such as mobile apps or online platforms, might be helpful to increase accessibility and to promote engagement in healthy lifestyle behaviours.

Eighth, lifestyle interventions in general have the potential to be provided at relatively low cost but given the often limited resources in local community settings, future studies should also include economic evaluations to provide convincing data on the economic benefits of lifestyle interventions in adults with SMI.

Finally, we should not only examine the short-term effects of lifestyle interventions, as it is currently most often the case due to feasibility constraints (study funding only for specific durations and lacking observational data on long-term uptake and efficacy), but also explore strategies for sustaining positive lifestyle changes and investigate their long-term impact on physical and mental health outcomes. Overall, addressing these research gaps will contribute to a more comprehensive understanding of how best to use lifestyle interventions.

Results from this guidance paper should be interpreted within its strengths and limitations. The strength of this guidance paper lies in its comprehensive approach covering several domains of the heterogeneous field of lifestyle interventions in SMI populations and its transparent methodology for evidence synthesis and recommendation development. However, summarizing the evidence and developing recommendations highlighted several challenges, arising mainly from the heterogeneity of the available evidence. Since our guidance paper is based on evidence from already existing systematic reviews, we have thus omitted primary studies that have not yet been included in reviews. In addition, we focused on the most common types of interventions related to physical activity, diet and sleep. Although these broad categories cover a wide spectrum of interventions, we left aside evidence from other emerging lifestyle domains, such as those targeting loneliness and social interactions, related to the workplace and working conditions, or stress management and mindfulness-based interventions. In some cases, we had to artificially categorise lifestyle behaviours that span several domains and may have effects at multiple levels within a single category. The interventions within each category also varied considerably, making it difficult to summarise them for the development of recommendations. Moreover, many factors might moderate the beneficial effects of lifestyle interventions, some of which are often under-reported. For example, the type of professional supervision, the size of the group and the individual adaption of the interventions might have a huge impact on results. These adaptations are particularly important to ensure applicability to special patient populations, such as those with physical disability or cognitive impairment. In some cases, lifestyle interventions might be generally unacceptable or inappropriate, so that medication options should be considered for specific outcomes, for example, metformin for antipsychotic-induced weight gain [151]. Future research should also assess these aspects in more detail and implement them in further recommendations. Finally, we focused on lifestyle interventions in adults with SMI. The degree to which the results can be transferred to children and adolescents living with SMI is not clear and requires further study and a separate review.

## Conclusions

This guidance paper provides a comprehensive summary of the current meta-review evidence regarding the benefits of lifestyle interventions in adults with SMI and identified current research gaps. Evidence supports the application of lifestyle interventions that combine behavioural change techniques, dietary modification, and physical activity to reduce weight and improve cardiovascular health parameters in adults with SMI. Our meta-review indicated a relative lack of research regarding interventions for adults with bipolar disorder, as well as on nutritional and sleep interventions. With further research, lifestyle interventions can and should be a core component of mental health care in the future.

## Supporting information

Maurus et al. supplementary material Maurus et al. supplementary material

## Supplementary material

The supplementary material for this article can be found at http://doi.org/10.1192/j.eurpsy.2024.1766.
