# Supplementary material for: EPA guidance on lifestyle interventions for adults with severe mental illness: A meta-review of the evidence
Source: Eur Psychiatry. 2024 Dec 10;67(1):e80. doi: 10.1192/j.eurpsy.2024.1766 (PMC11733621; doi:10.1192/j.eurpsy.2024.1766)
Supplement: Maurus et al. supplementary material [file S0924933824017668sup001.docx]

**Supplement**

**S1: Detailed search strategies for the different databases**

### S1.1: Search strategy PubMed

### (“bipolar disorder”[mh] OR “depressive disorder, major”[mh] OR “schizophrenia spectrum and other psychotic disorders”[mh] OR “major depress*“[tiab] OR bipolar[tiab] OR schizophren*[tiab] OR “severe mental illness*”[tiab] OR “serious mental illness*”[tiab] OR “severe mental disorder*”[tiab] OR “serious mental disorder*”[tiab] AND ((“health behavior”[mh] OR “lifestyle intervention*”[tiab] OR “lifestyle medicine”[tiab] OR “lifestyle psychiatry”[tiab]) OR (“weight loss”[mh] OR “weight reduction programs”[mh] OR “diet therapy”[mh] OR “interventions for weight loss”[tiab] OR “weight management”[tiab] OR diet*[tiab] OR nutrition*[tiab]) OR ((exercise[mh] OR exercis*[tiab] OR “physical* activ*”[tiab] OR sport*[tiab] OR “yoga”[mh] OR yoga[tiab]) AND 2018/01/16:2022 [dp]) OR (“sleep hygiene”[mh] OR “sleep quality”[tiab] OR “improve sleep”[tiab] OR “sleep intervention*”[tiab] OR “sleep hygiene”[tiab])) AND (guideline[pt] OR systematic review[pt] OR meta-analysis[pt] OR systematic[sb] OR meta-analysis[tiab]) AND Humans[filter]

### S1.2: Search strategy EMBASE

Check box “Map Term to Subject Headings” for each term

(exp Bipolar Disorder/ OR exp Depressive Disorder, Major/ OR exp Schizophrenia/ OR exp Psychotic Disorders/ OR major depress*.ti,ab OR bipolar.ti,ab OR schizophren*.ti,ab OR severe mental illness*.ti,ab OR serious mental illness*.ti,ab OR severe mental disorder*.ti,ab OR serious mental disorder*.ti,ab) AND ((exp Health Behavior/ OR lifestyle intervention*.ti,ab OR lifestyle medicine.ti,ab OR lifestyle psychiatry.ti,ab) OR (exp Weight Loss/ OR exp Weight Reduction Programs/ OR exp Diet Therapy/ OR interventions for weight loss.ti,ab OR weight management.ti,ab OR di-et*.ti,ab OR nutrition*.ti,ab) OR (exp Exercise/ OR exp Exercise Therapy/ OR exp Sports/ OR exp Yoga/ OR exercis*.ti,ab OR physical* activ*.ti,ab OR sport*.ti,ab OR yoga.ti,ab)) OR (exp Sleep Hygiene/ OR sleep quality.ti,ab OR improve sleep.ti,ab OR sleep intervention*.ti,ab OR sleep hygiene.ti,ab)) AND (exp Practice Guideline/ OR exp "Systematic Review"/ OR exp Meta-Analysis/ OR systematic review.ti,ab OR meta-analysis.ti,ab OR guideline.ti,ab) AND limit (exp Exercise/…) to yr=”2018-2022” AND limit (humans)

### S1.3: Search strategy Cochrane Database for Systematic Reviews

(“bipolar disorder”:ti,ab OR “schizophrenia spectrum and other psychotic disorders”:ti,ab OR (major NEXT depress*):ti,ab OR bipolar:ti,ab OR schizo-phren*:ti,ab OR (severe NEXT mental NEXT illness*):ti,ab OR (serious NEXT mental NEXT illness*):ti,ab OR (severe NEXT mental NEXT disorder*):ti,ab OR (serious NEXT mental NEXT disorder*):ti,ab) AND (“health behavior”:ti,ab OR (lifestyle NEXT intervention*):ti,ab OR “lifestyle medicine”:ti,ab OR “lifestyle psychiatry”:ti,ab) OR (“weight loss”:ti,ab OR (weight reduction program*):ti,ab OR “diet therapy”:ti,ab OR “interventions for weight loss”:ti,ab OR “weight management”:ti,ab OR diet*:ti,ab OR nutrition*:ti,ab) OR (exercis*:ti,ab OR (physical* NEXT activ*):ti,ab OR sport*:ti,ab OR yoga:ti,ab) OR (“sleep hygiene”:ti,ab OR “sleep quality”:ti,ab OR “improve sleep”:ti,ab OR (sleep NEXT intervention*):ti,ab) AND (guideline:pt OR “systematic review”:pt OR meta-analysis:pt OR “systematic review”:ti,ab OR meta-analysis:ti,ab)

### S1.4: Search strategy Epistemonikos

(title:(major depress*) OR abstract:(major depress*) OR title:(bipolar) OR abstract:(bipolar) OR title:(schizophren*) OR abstract:(schizophren*) OR ti-tle:(severe mental illness*) OR abstract:(severe mental illness*) OR title:(serious mental illness*) OR abstract:(serious mental illness*) OR title:(severe men-tal disorder*) OR abstract:(severe mental disorder*) OR title:(serious mental disorder*) OR abstract:(serious mental disorder*)) AND ((title:(lifestyle intervention*) OR abstract:(lifestyle intervention*) OR title:”lifestyle medicine” OR abstract:”lifestyle medicine” OR title:”lifestyle psychiatry” OR abstract:”lifestyle psychiatry”) OR (title:”weight loss” OR abstract:”weight loss” OR title:“weight reduction programs” OR abstract:“weight reduction programs” OR title:“diet therapy” OR ab-stract:“diet therapy” OR title:“interventions for weight loss” OR abstract:“interventions for weight loss” OR title:“weight management” OR abstract:“weight management” OR title:diet* OR abstract:diet* OR title:nutrition* OR abstract:nutrition*) OR (title:exercis* OR abstract:exercis* OR title:(physical* activ*) OR abstract:(physical* activ*) OR title:sport* OR abstract:sport* OR title:”yoga” OR ab-stract:”yoga”) OR (title:”sleep hygiene” OR abstract:”sleep hygiene” OR title:“sleep quality” OR abstract:“sleep quality” OR title:“improve sleep” OR abstract:“improve sleep” OR title:(sleep intervention*) OR abstract:(sleep intervention*))) AND (title:”systematic review” OR abstract:”systematic review” OR title:meta-analysis OR abstract:meta-analysis)

Note: Date of publication not available for single terms (physical activity/exercise); humans filter not available; language filter not available.

**S2: Inclusion and exclusion criteria**

|  | **Inclusion and exclusion criteria** | |
| --- | --- | --- |
|  | **Inclusion** | **Exclusion** |
| **Population** - Adults with severe mental illnesses | | |
| Adults | ≥ 18 years | < 18 years |
| Severe  mental  illness | - schizophrenia spectrum disorders - major depressive disorder - bipolar disorder - with or without pre-existing cardiopulmonary and cardiovascular comorbidity or metabolic syndrome | - other mental disorders - depressive disorders other than major depressive disorder (e. g. seasonal depression, premenstrual dysphoric disorder, post-partum depression) - peri- and post-partum severe mental illnesses - patients in palliative care - systematic reviews of studies in which <50% of participants in the interventional groups had any of the mental disorders under review - animal models |
| **Intervention** - Lifestyle interventions (LI) | | |
| Interventions  in general | - non-pharmacological lifestyle interventions related to the treatment of mental disorders - targeting physical activity (incl. yoga, tai chi, qigong), weight, diet or sleep - including psychoeducational and/or cognitive-behavioural interventions - multimodal lifestyle interventions (including the above mentioned) | - pharmacological interventions - interventions regarding stress management, e. g., meditation or mindfulness - diet interventions solely focusing on the intake of specific preparations, e. g., supplements or vitamins - interventions involving the passive use of objects such as bright light lamps or blue-blocking glasses |
| Setting | - inpatient or outpatient setting - delivered individually or group based | --- |
| **Comparison intervention** - Not defined | | |
| Control group | - any control condition, e. g., treatment as usual, active control group, no treatment or waiting list | --- |
| **Outcomes** - Broadly defined | | |
| Health  behaviors | - factors considered as health behaviors, e. g., amount of physical activity | --- |
| Cardiovascular  risk factors | - factors considered as cardiovascular risk factors, e. g. hypertonia or obesity | --- |
| Psychopathol-ogy measures | - factors associated with psychopathology, e. g., cognitive functioning, remission or relapse | - mechanistic factors of psychopathology, e. g., brain structural changes |
| Quality of life/  functional outcomes | - assessments of the patients’ quality of life or level of functioning in daily life | --- |
| **Study type** - Systematic review | | |
| Systematic  review | - systematic review (with or without a meta-analysis) that assessed studies of any design [interventional (experimental, quasi-experimental, pre-experimental), observational and case studies] | - conference abstracts - studies that were meta-reviews or guidelines that did not search systematically |
| **Time period of search** | | |
| Dates of search | - 16^th^ September 2022: Date of search for PubMed, Cochrane Database of Systematic Reviews and Epistemonikos - 7^th^ October 2022: Date of search for EMBASE - 16^th^ January 2018 until dates of search: Reviews related to physical activity, exercise and sports | - physical activity, exercise and sports: until 15/01/2018 |

**S3: Excluded Full-texts**

| **Reference** | **Reason for exclusion** |
| --- | --- |
| Allison DJ, Sharma B, Timmons BW. The efficacy of anti-inflammatory treatment interventions on depression in individuals with major depressive disorder and high levels of inflammation: A systematic review of randomized clinical trials. Physiology & behavior. 2019;207:104-12.  doi: 10.1016/j.physbeh.2019.05.006 | Review on physical activity including only studies published until Jan 15th 2018 |
| Andrade C. Cardiometabolic Risks in Schizophrenia and Directions for Intervention, 2: Nonpharmacological Interventions. J Clin Psychiatry. 2016 Aug;77(8):e964-7.  doi: 10.4088/JCP.16f11060. PMID: 27561152. | Wrong type of review, e.g. meta-review, narrative review |
| Ashdown-Franks G, Firth J, Carney R, Carvalho AF, Hellgren M, Koyanagi A et al. Exercise as Medicine for Mental and Substance Use Disorders: A Meta-review of the Benefits for Neuropsychiatric and Cognitive Outcomes. Sports Med 50, 151–170 (2020).  https://doi.org/10.1007/s40279-019-01187-6 | Wrong type of review, e.g. meta-review, narrative review |
| Asher GN, Gartlehner G, Gaynes BN, Amick HR, Forneris C, Morgan LC, Coker-Schwimmer E, Boland E, Lux LJ, Gaylord S, Bann C, Pierl CB, Lohr KN. Comparative Benefits and Harms of Complementary and Alternative Medicine Therapies for Initial Treatment of Major Depressive Disorder: Systematic Review and Meta-Analysis. J Altern Complement Med. 2017 Dec;23(12):907-919. doi: 10.1089/acm.2016.0261. Epub 2017 Jul 12. PMID: 28700248. | Review on physical activity including only studies published until Jan 15th 2018 |
| Bell PF, McKenna JP, Roscoe BM. Treatment of bipolar disorders and metabolic syndrome: implications for primary care. Postgraduate Medicine (2009), 121(5), 140-144. | Interventions other than predefined lifestyle interventions |
| Bonfioli E, Berti L, Goss C, Muraro F, Burti L. Health promotion lifestyle interventions for weight management in psychosis: a systematic review and meta-analysis of randomised controlled trials. BMC Psychiatry. 2012 Jul 12;12:78. doi: 10.1186/1471-244X-12-78. PMID: 22789023; PMCID: PMC3549787. | Diagnoses other than predefined SMI and/or no subgroup analyses available |
| Bradley T, Campbell E, Dray J, Bartlem K, Wye P, Hanly G et al. Systematic review of lifestyle interventions to improve weight, physical activity and diet among people with a mental health condition. Syst Rev 11, 198 (2022). https://doi.org/10.1186/s13643-022-02067-3. | Review on physical activity including only studies published until Jan 15th 2018 |
| Baxter A, Harris M, Khatib Y, Brugha T, Bien H, Bhui K. Reducing excess mortality due to chronic disease in people with severe mental illness: Meta-review of health interventions. British Journal of Psychiatry, 208(4), 322-329 (2016). doi:10.1192/bjp.bp.115.163170 | Review on physical activity including only studies published until Jan 15th 2018 |
| Brietzke E, Cerqueira RO, Mansur, Rodrigo BM, McIntyre RS. GLUTEN RELATED ILLNESS AND SEVERE MENTALDISORDERS: A COMPREHENSIVE REVIEW.Neuroscience and BiobehavioralReviews (2017). https://doi.org/10.1016/j.neubiorev.2017.08.009 | Interventions other than predefined lifestyle interventions |
| Broderick J, Vancampfort D. Yoga as part of a package of care versus non-standard care for schizophrenia. Cochrane Database of Systematic Reviews 2019, Issue 4. Art. No.: CD012807. DOI:10.1002/14651858. CD012807. pub2. | Review on physical activity including only studies published until Jan 15th 2018 |
| Brondino N, Rocchetti M, Fusar-Poli L, Codrons E, Correale L, Vandoni M, Barbui C, Politi P. A systematic review of cognitive effects of exercise in depression. Acta Psychiatr Scand. 2017 Apr;135(4):285-295. doi: 10.1111/acps.12690. Epub 2017 Jan 22. PMID: 28110494. | Review on physical activity including only studies published until Jan 15th 2018 |
| Bueno-Antequera J, Munguía-Izquierdo D. Exercise and Schizophrenia. In: Xiao, J. (eds) Physical Exercise for Human Health. Advances in Experimental Medicine and Biology, vol 1228 (2020). Springer, Singapore. https://doi.org/10.1007/978-981-15-1792-1_21 | Wrong type of review, e.g. meta-review, narrative review |
| Burton A, Walters K, Atkins L, Howard M, Michie S, Peveler R et al. Barriers, facilitators, and effective interventions for lowering cardiovascular disease risk in people with severe mental illnesses: evidence from a systematic review and focus group study. The Lancet, 388, S30 (2016). | No full text found |
| Cabassa LJ, Ezell JM, Lewis-Fernández R. Lifestyle interventions for adults with serious mental illness: a systematic literature review. Psychiatric services (Washington, D.C.), 61(8), 774–782 (2010). https://doi.org/10.1176/ps.2010.61.8.774 | Diagnoses other than predefined SMI and/or no subgroup analyses available |
| Conn S, Curtain S. Health coaching as a lifestyle medicine process in primary care. Aust J Gen Pract. 2019 Oct;48(10):677-680. doi: 10.31128/AJGP-07-19-4984. PMID: 31569315. | Wrong type of review, e.g. meta-review, narrative review |
| Cooney GM, Dwan K, Greig CA, et al. Exercise for depression. Cochrane Database Syst Rev. 2013;12(9):CD004366 | Review on physical activity including only studies published until Jan 15th 2018 |
| Cramer H, Lauche R, Klose P, Langhorst J, Dobos G. Yoga for schizophrenia: A systematic review and meta-analysis. BMC Psychiatry. 2013;13:32. | Review on physical activity including only studies published until Jan 15th 2018 |
| Cramer H, Anheyer D, Lauche R, Dobos GA. Systematic review of yoga for major depressive disorder. J Affect Disord. 2017 Apr 15;213:70-77. doi: 10.1016/j.jad.2017.02.006. Epub 2017 Feb 7. PMID: 28192737 | Review on physical activity including only studies published until Jan 15th 2018 |
| Czosnek L, Lederman O, Cormie P, Zopf E, Stubbs B, Rosenbaum S. Health benefits, safety and cost of physical activity interventions for mental health conditions:A meta-review to inform translation efforts, Mental Health and Physical Activity (2018), doi: https://doi.org/10.1016/j.mhpa.2018.11.001. | Review on physical activity including only studies published until Jan 15th 2018 |
| DÁmico L, Jaffe LE, Gardner JA. (2018). Evidence for Interventions to improve and maintain occupational performance and participation for people with serious mental illness: a systematic review. Am J Occup Ther. 2018 Sep/Oct;72(5):7205190020p1-7205190020p11. PMID: 30157006 DOI: 10.5014/ajot.2018.033332. | Interventions other than predefined lifestyle interventions |
| Danielsson L, Noras AM, Waern M, Carlsson J. Exercise in the treatment of major depression: a systematic review grading the quality of evidence. Physiother Theory Pract. 2013 Nov;29(8):573-85. DOI: 10.3109/09593985.2013.774452. Epub 2013 Mar 22.PMID: 23521569. | Review on physical activity including only studies published until Jan 15th 2018 |
| Dauwan M, Begemann MJ, Heringa SM, Sommer IE. Exercise Improves Clinical Symptoms, Quality of Life, Global Functioning, and Depression in Schizophrenia: A Systematic Review and Meta-analysis. Schizophr Bull. 2016 May;42(3):588-99. doi: 10.1093/schbul/sbv164. Epub 2015 Nov 7. PMID: 26547223; PMCID: PMC4838091. | Review on physical activity including only studies published until Jan 15th 2018 |
| Dauwan M, Begemann MJH, Slot MIE et al. Physical exercise improves quality of life, depressive symptoms, and cognition across chronic brain disorders: a transdiagnostic systematic review and meta-analysis of randomized controlled trials. J Neurol 268, 1222–1246 (2021). https://doi.org/10.1007/s00415-019-09493-9 | Diagnoses other than predefined SMI and/or no subgroup analyses available |
| Dixon LB, Dickerson F, Bellack AS, Bennett, M., Dickinson D, Goldberg RW et al. The 2009 Schizophrenia PORT Psychosocial Treatment Recommendations and Summary Statements. Schizophr Bull. 2010 Jan;36(1):48-70. doi: 10.1093/schbul/sbp115. Epub 2009 Dec 2. PMID: 19955389 PMCID: PMC2800143. | Wrong type of review, e.g. meta-review, narrative review |
| Faulkner S, Bee P. Perspectives on Sleep, Sleep Problems, and Their Treatment, in People with Serious Mental Illnesses: A Systematic Review (2016). PLoS ONE 11(9): e0163486. doi:10.1371/ journal.pone.0163486 | Interventions other than predefined lifestyle interventions |
| Fernández-San-Martín MI, Martín-López LM, Masa-Font R, Olona-Tabueña N, Roman Y, Martin-Royo J et al. The effectiveness of lifestyle interventions to reduce cardiovascular risk in patients with severe mental disorders: meta-analysis of intervention studies. Community Ment Health J. 2014 Jan;50(1):81-95. doi: 10.1007/s10597-013-9614-6. Epub 2013 Jun 6. PMID: 23739948. | (Part of the) sample < age of 18 |
| Fink A, Cieslak S, Rosenbach F. Nichtpharmakologische Interventionen zur Prävention von Gewichtszunahme bei schizophrenen Patienten unter antipsychotischer Medikation [Non-Pharmacological Interventions for the Prevention of Weight Gain in Schizophrenic Patients Treated with Antipsychotic Medication]. Psychiatr Prax. 2015 Oct;42(7):359-69. German. doi: 10.1055/s-0035-1552670. Epub 2015 Jul 9. PMID: 26158719. | Full text article not published in English |
| Finnell D, Ditz KA. Health diaries for self-monitoring and self-regulation: applications to individuals with serious mental illness. Issues Ment Health Nurs. 2007 Dec;28(12):1293-307. doi: 10.1080/01612840701686435. PMID: 18058335. | Review on physical activity including only studies published until Jan 15th 2018 |
| Firth J, Marx W, Dash S, Carney R, Teasdale SB, Solmi M et al. The effects of dietary improvements on symptoms of depression and anxiety: A meta-analysis of randomised controlled trials. Psychosom Med 2019;81(3):265–80. doi: 10.1097/PSY.0000000000000673. | Diagnoses other than predefined SMI and/or no subgroup analyses available |
| Fortuna K, Naslund J, LaCroix J, Bianco C, Brooks J, Zisman-Ilani Y, Muralidharan A, Deegan P Digital Peer Support Mental Health Interventions for People With a Lived Experience of a Serious Mental Illness: Systematic Review JMIR Ment Health 2020;7(4):e16460 URL: https://mental.jmir.org/2020/4/e16460 DOI: 10.2196/16460 | Outcome measures other than psychopathology, cognitive functioning, quality of life or changes in physical health indicators |
| Gabriele JM, Dubbert PM, Reeves RR. Efficacy of behavioural interventions in managing atypical antipsychotic weight gain. Obesity Reviews, 10: 442-455 (2009). https://doi.org/10.1111/j.1467-789X.2009.00570.x | Diagnoses other than predefined SMI and/or no subgroup analyses available |
| Gassner L, Dabnichki P, Langer A, Pokan R, Zach H, Ludwig M, Santer A. The Therapeutic Effects of Climbing: A Systematic Review and Meta-Analysis. PM&R: The Journal of Injury, Function and Rehabilitation (2022). Accepted Author Manuscript. https://doi.org/10.1002/pmrj.12891 | Diagnoses other than predefined SMI and/or no subgroup analyses available |
| Gorczynski P, Faulkner G. Exercise therapy for schizophrenia. Cochrane Database Syst Rev. 2010 May 12;(5):CD004412. doi: 10.1002/14651858.CD004412.pub2. PMID: 20464730; PMCID: PMC4164954. | Review on physical activity including only studies published until Jan 15th 2018 |
| Gronholm PC, Chowdhary N, Barbui C, Das-Munshi J, Kolappa K, Thornicroft G et al.. Prevention and management of physical health conditions in adults with severe mental disorders: WHO recommendations. Int J Ment Health Syst 15, 22 (2021). https://doi.org/10.1186/s13033-021-00444-4 | Wrong type of review, e.g. meta-review, narrative review |
| Gross J, Vancampfort D, Stubbs B, Gorczynski P, Soundy, A. A narrative synthesis investigating the use and value of social support to promote physical activity among individuals with schizophrenia. Disability and rehabilitation, 38(2), 123-150 (2016). | Review on physical activity including only studies published until Jan 15th 2018 |
| Gurusamy J, Gandhi S, Damodharan D, Ganesan V, Palaniappan M. Exercise, diet and educational interventions for metabolic syndrome in persons with schizophrenia: A systematic review. Asian J Psychiatr. 2018 Aug;36:73-85. doi: 10.1016/j.ajp.2018.06.018. Epub 2018 Jun 30. PMID: 29990631. | Review on physical activity including only studies published until Jan 15th 2018 |
| Gühne U, Weinmann S, Riedel-Heller SG, Becker T. Psychosocial therapies in severe mental illness: update on evidence and recommendations. Current Opinion in Psychiatry 33(4):p 414-421, July 2020. \| DOI: 10.1097/YCO.0000000000000618 | Wrong type of review, e.g. meta-review, narrative review |
| Hanstock T, Speirs B, Kay-Lambkin F. A systematic review of evidence for lifestyle interventions in people with bipolar disorder. In Bipolar Disorders (Vol. 22, pp. 100-100). 111 RIVER ST, HOBOKEN 07030-5774, NJ USA: WILEY (2020). | No full text found |
| Hanstock T, Speirs B, Kay-Lambkin F. A large systematic review of evidence for lifestyle interventions targeting smoking, sleep, alcohol/other drug use, physical activity and healthy diet in people with bipolar disorder. In Bipolar Disorders (Vol. 23, pp. 49-49). 111 RIVER ST, HOBOKEN 07030-5774, NJ USA: WILEY (2021). | No full text found |
| Hess CW, Karter J, Cosgrove L, Hayden L. Evidence-based practice: a comparison of International Clinical Practice Guidelines and current research on physical activity for mild to moderate depression. Transl Behav Med. 2019 Jul 16;9(4):703-710. doi: 10.1093/tbm/iby092. PMID: 30321410. | Outcome measures other than psychopathology, cognitive functioning, quality of life or changes in physical health indicators |
| Hester L, Dang D, Barker CJ, Heath M, Mesiya S, Tienabeso T, Watson K. Evening wear of blue-blocking glasses for sleep and mood disorders: a systematic review. Chronobiol Int. 2021 Oct;38(10):1375-1383. doi: 10.1080/07420528.2021.1930029. Epub 2021 May 24. PMID: 34030534. | Diagnoses other than predefined SMI and/or no subgroup analyses available |
| Heywood SE, Connaughton J, Kinsella R, Black S, Bicchi N, Setchell J. Physical Therapy and Mental Health: A Scoping Review. Physical Therapy, 102(11), pzac102 (2022). | Wrong type of review, e.g. meta-review, narrative review |
| Holley J, Crone D, Tyson P, Lovell G. The effects of physical activity on psychological well-being for those with schizophrenia: A systematic review. Br J Clin Psychol. 2011;50(1):84–105 | Review on physical activity including only studies published until Jan 15th 2018 |
| Hui TT, Garvey L, Olasoji M. Improving the physical health of young people with early psychosis with lifestyle interventions: Scoping review. Int J Mental Health Nurs, 30: 1498-1524 (2021). https://doi.org/10.1111/inm.12922 | (Part of the) sample < age of 18 |
| Ilyas A, Chesney E, Patel R. Does physical health monitoring reduce mortality in people with psychotic disorders? European Psychiatry, 48(Supplement), S237 (2018). [PW0263]. | Wrong type of review, e.g. meta-review, narrative review |
| Ismail K. Physical health in schizophrenia and related disorders. Psychiatry, 7(11), 472-476 (2008). | Wrong type of review, e.g. meta-review, narrative review |
| Jean M, Umair M, Muddaloor P, Farinango M, Ansary A, Dakka A, Nazir Z, Shamim H, Paidi G, Khan S. The Effects of Yoga on Bipolar Disorder: A Systematic Review. Cureus.2022; 14(8), e27688. https://doi.org/10.7759/cureus.27688 | Wrong type of review, e.g. meta-review, narrative review |
| Jormfeldt H, Carlsson IM. Equine-Assisted Therapeutic Interventions Among Individuals Diagnosed With Schizophrenia. A Systematic Review. Issues Ment Health Nurs. 2018 Aug;39(8):647-656. doi: 10.1080/01612840.2018.1440450. Epub 2018 Mar 6. PMID: 29509053. | Review on physical activity including only studies published until Jan 15th 2018 |
| Keller-Varady K, Varady PA, Röh A, Schmitt A, Falkai P, Hasan A, Malchow B. A systematic review of trials investigating strength training in schizophrenia spectrum disorders. Schizophr Res. 2018 Feb;192:64-68. doi: 10.1016/j.schres.2017.06.008. Epub 2017 Aug 24. PMID: 28602648. | Review on physical activity including only studies published until Jan 15th 2018 |
| Kieckhaefer C, Broer K, Theisen C. Relaxation techniques for psychosis treatment. E-Poster Viewing. (2019). European Psychiatry, 56(S1), S322-S553. doi:10.1016/j.eurpsy.2019.01.002 | Wrong type of review, e.g. meta-review, narrative review |
| Korman N, Chapman JJ, Firth J, Armour M, Suetani S, Rosenbaum S, Vancampfort D, Stubbs B, Siskind D. Effects of high intensity interval training in people with severe mental illness: A systematic review of intervention studies-considering diverse approaches to improving mental and physical health outcomes. RANZCP Abstracts. Australian & New Zealand Journal of Psychiatry. 53(1_suppl):3-156 (2019). doi:10.1177/0004867419836919 | Review on physical activity including only studies published until Jan 15th 2018 |
| Kovacevic A, Mavros Y, Heisz JJ, Fiatarone Singh MA. The effect of resistance exercise on sleep: A systematic review of randomized controlled trials. Sleep Med Rev. 2018 Jun;39:52-68. doi: 10.1016/j.smrv.2017.07.002. Epub 2017 Jul 19. PMID: 28919335. | Review on physical activity including only studies published until Jan 15th 2018 |
| Kozumplik O, Uzun S, Jakovljević M. Metabolic syndrome in patients with psychotic disorders: diagnostic issues, comorbidity and side effects of antipsychotics. Psychiatr Danub. 2010 Mar;22(1):69-74. PMID: 20305594. | Wrong type of review, e.g. meta-review, narrative review |
| Kvam S, Kleppe CL, Nordhus IH, Hovland A. Exercise as a treatment for depression: A meta-analysis. J Affect Disord. 2016 Sep 15;202:67-86. doi: 10.1016/j.jad.2016.03.063. Epub 2016 May 20. PMID: 27253219. | Review on physical activity including only studies published until Jan 15th 2018 |
| Lambert TJ, Chapman LH. Consensus Working Group. Diabetes, psychotic disorders and antipsychotic therapy: a consensus statement. Med J Aust. 2004 Nov 15;181(10):544-8. doi: 10.5694/j.1326-5377.2004.tb06443.x. PMID: 15540966. | Wrong type of review, e.g. meta-review, narrative review |
| Lederman O, Ward PB, Firth J, Maloney C, Carney R, Vancampfort D et al. Does exercise improve sleep quality in individuals with mental illness? A systematic review and meta-analysis. J Psychiatr Res. 2019 Feb;109:96-106. doi: 10.1016/j.jpsychires.2018.11.004. Epub 2018 Nov 3. PMID: 30513490. | Diagnoses other than predefined SMI and/or no subgroup analyses available |
| Levinta A, Mukovozov I, Tsoutsoulas C. Use of a Gluten-Free Diet in Schizophrenia: A Systematic Review. Adv Nutr. 2018 Nov 1;9(6):824-832. doi: 10.1093/advances/nmy056. PMID: 30325398; PMCID: PMC6247287. | (Part of the) sample < age of 18 |
| Li J, Shen J, Wu G, Tan Y, Sun Y, Keller E et al. Mindful exercise versus non-mindful exercise for schizophrenia: A systematic review and meta-analysis of randomized controlled trials. Complement Ther Clin Pract. 2018 Aug;32:17-24. doi: 10.1016/j.ctcp.2018.04.003. Epub 2018 Apr 7. PMID: 30057047. | Review on physical activity including only studies published until Jan 15th 2018 |
| Lin K, Tao L. Exercise on bipolar disorder in humans. Int Rev Neurobiol. 2019;147:189-198. doi: 10.1016/bs.irn.2019.07.001. Epub 2019 Jul 26. PMID: 31607354 | Wrong type of review, e.g. meta-review, narrative review |
| Manger S. Lifestyle interventions for mental health. Aust J Gen Pract. 2019 Oct;48(10):670-673. doi: 10.31128/AJGP-06-19-4964. PMID: 31569326. | Wrong type of review, e.g. meta-review, narrative review |
| Manu P, Dima L, Shulman M, Vancampfort D, De Hert M, Correll CU. Weight gain and obesity in schizophrenia: epidemiology, pathobiology, and management. Acta Psychiatrica Scandinavica, 132(2), 97-108 (2015). | Wrong type of review, e.g. meta-review, narrative review |
| Marquez DX, Aguiñaga S, Vásquez PM, Conroy DE, Erickson KI, Hillman C et al. A systematic review of physical activity and quality of life and well-being. Transl Behav Med. 2020 Oct 12;10(5):1098-1109. doi: 10.1093/tbm/ibz198. PMID: 33044541; PMCID: PMC7752999. | Review on physical activity including only studies published until Jan 15th 2018 |
| Martens N, Destoop M, Dom G. Organization of Community Mental Health Services for Persons with a Severe Mental Illness and Comorbid SomaticConditions: A Systematic Review on Somatic Outcomesand Health Related Quality of Life. Int J Environ Res Public Health. 2021 Jan 8;18(2):462. PMID: 33435525. PMCID: PMC7826863. doi: 10.3390/ijerph18020462. | Interventions other than predefined lifestyle interventions |
| Martland R, Mondelli V, Gaughran V , Stubbs B. Can high intensity interval training improve health outcomes among people with mental illness? A sys- tematic review and preliminary meta-analysis of intervention studies across a range of mental illnesses, Journal of Affective Disorders (2019), doi: https://doi.org/10.1016/j.jad.2019.11.039 | Diagnoses other than predefined SMI and/or no subgroup analyses available |
| Martland R, Stubbs B, Gaughran F. T147. Can high intensity interval training (HIIT) improve physical and mental health outcomes? A metareview of the global benefits of hiit and focused systematic review of the effects of hiit in mental disorders, Schizophrenia Bulletin, Volume 46, Issue Supplement_1, April 2020, Pages S286–S287, https://doi.org/10.1093/schbul/sbaa029.707 | Wrong type of review, e.g. meta-review, narrative review |
| Mclntyre R,S. Review: non-pharmacological interventions reduce antipsychotic induced weight gain. Evid Based Ment Health. 2009 May;12(2):52. doi: 10.1136/ebmh.12.2.52. PMID: 19395611. | Wrong type of review, e.g. meta-review, narrative review |
| Meister K, Juckel G. A Systematic Review of Mechanisms of Change in Body-Oriented Yoga in Major Depressive Disorders. (2017). DOIhttps://doi.org/10.1055/s-0043-111013. | Review on physical activity including only studies published until Jan 15th 2018 |
| Meyer J. Treating the Mind and Body in Schizophrenia: Risks and Prevention. CNS Spectrums, 9(S11), 25-33 (2004). doi:10.1017/S1092852900025104 | Wrong type of review, e.g. meta-review, narrative review |
| Miller KJ, Gonc ̧alves-Bradley DC, Areerob P, Hennessy D, MesagnoC, Grace F. Comparative Effectiveness of Three Exercise Types to Treat Clinical Depressionin Older Adults: A Systematic Review and Network Meta-Analysis of Randomised ControlledTrials,Ageing Research Reviews(2019), doi:https://doi.org/10.1016/j.arr.2019.100999 | Review on physical activity including only studies published until Jan 15th 2018 |
| Mishu MP, Uphoff E, Aslam F, Philip S, Wright J, Tirbhowan N, et al. Interventions for preventing type 2 diabetes in adults with mental disorders in low- and middle-income countries. The Cochrane database of systematic reviews. 2021;2(2):Cd013281. | Interventions other than predefined lifestyle interventions |
| Morgan AJ, Jorm AF. Self-help interventions for depressive disorders and depressive symptoms: a systematic review. Ann Gen Psychiatry. 2008 Aug 19;7:13. doi: 10.1186/1744-859X-7-13. PMID: 18710579; PMCID: PMC2542367. | Review on physical activity including only studies published until Jan 15th 2018 |
| Morres ID, Hatzigeorgiadis A, Stathi A, Comoutos N, Arpin-Cribbie C, Krommidas C et al. Aerobic exercise for adult patients with major depressive disorder in mental health services: A systematic review and meta-analysis. Depress Anxiety. 2019 Jan;36(1):39-53. doi: 10.1002/da.22842. Epub 2018 Oct 18. PMID: 30334597. | Review on physical activity including only studies published until Jan 15th 2018 |
| Newcomer, J. Elevating the standard of care in the management of cardiometabolic risk factors in patients with mental illness. CNS Spectrums, 13(6), 1-14(2008). | Wrong type of review, e.g. meta-review, narrative review |
| Nyboe L, Lemcke S, Møller AV, Stubbs B. Non-pharmacological interventions for preventing weight gain in patients with first episode schizophrenia or bipolar disorder: A systematic review. Psychiatry Res. 2019 Nov;281:112556. doi: 10.1016/j.psychres.2019.112556. Epub 2019 Sep 4. PMID: 31521840. | (Part of the) sample < age of 18 |
| Olagunju AE, Gaddey H. Leveraging CAM to treat depression and anxiety. J Fam Pract. 2020 Jun;69(5):221-227. PMID: 32555752. | Wrong type of review, e.g. meta-review, narrative review |
| Opie RS, O'Neil A, Itsiopoulos C, Jacka FN. The impact of whole-of-diet interventions on depression and anxiety: a systematic review of randomised controlled trials. Public Health Nutr. 2015 Aug;18(11):2074-93. doi: 10.1017/S1368980014002614. Epub 2014 Dec 3. PMID: 25465596. | Diagnoses other than predefined SMI and/or no subgroup analyses available |
| Papanastasiou E. Interventions for the metabolic syndrome in schizophrenia: a review. Ther Adv Endocrinol Metab. 2012 Oct;3(5):141-62. doi: 10.1177/2042018812458697. PMID: 23185687; PMCID: PMC3498847. | Interventions other than predefined lifestyle interventions |
| Pearsall R, Smith DJ, Pelosi A, Geddes J. Exercise therapy in adults with serious mental illness: a systematic review and meta-analysis. BMC Psychiatry. 2014 Apr 21;14:117. doi: 10.1186/1471-244X-14-117. PMID: 24751159; PMCID: PMC4018503. | Review on physical activity including only studies published until Jan 15th 2018 |
| Quirk H, Hock E, Harrop D, Crank H, Peckham E, Traviss-Turner G, Machaczek K, Stubbs B, Horspool M, Weich S, Copeland R. Understanding the experience of initiating community-based group physical activity by people with serious mental illness: A systematic review using a meta-ethnographic approach. Eur Psychiatry. 2020 Oct 22;63(1):e95. doi: 10.1192/j.eurpsy.2020.93. PMID: 33087211; PMCID: PMC7681136. | Review on physical activity including only studies published until Jan 15th 2018 |
| Ravindran AV, da Silva TL. Complementary and alternative therapies as add-on to pharmacotherapy for mood and anxiety disorders: a systematic review. J Affect Disord. 2013 Sep 25;150(3):707-19. doi: 10.1016/j.jad.2013.05.042. Epub 2013 Jun 12. PMID: 23769610. | Review on physical activity including only studies published until Jan 15th 2018 |
| Richter D, Gühne U, Stein J, Weinmann S, Becker T, Riedel-Heller SG. Die Effekte nicht-pharmakologischer, gesundheitsfördernder Interventionen bei Menschen mit schweren psychischen Erkrankungen [The Impact of Non-Pharmacologic Lifestyle Interventions in People with Severe Mental Illness - A Systematic Review]. Psychiatr Prax. 2018 Oct;45(7):347-357. German. doi: 10.1055/a-0677-7301. Epub 2018 Oct 10. PMID: 30304753. | Full text article not published in English |
| Riedel P, Smolka MN, Bauer M. Schizophrenie und bipolare Störung : Behandlung kognitiver Beeinträchtigungen [Schizophrenia and bipolar disorder : Treatment of cognitive impairments]. Nervenarzt. 2018 Jul;89(7):784-795. German. doi: 10.1007/s00115-018-0500-x. PMID: 29536121. | Full text article not published in English |
| Romain AJ, Bernard P, Akrass Z, St-Amour S, Lachance JP, Hains-Monfette G, Atoui S, Kingsbury C, Dubois E, Karelis AD, Abdel-Baki A. Motivational theory-based interventions on health of people with several mental illness: A systematic review and meta-analysis. Schizophr Res. 2020 Aug;222:31-41. doi: 10.1016/j.schres.2020.05.049. Epub 2020 Jun 7. PMID: 32522465. | Diagnoses other than predefined SMI and/or no subgroup analyses available |
| Roshanaei-Moghaddam B, Katon WJ, Russo J. The longitudinal effects of depression on physical activity. Gen Hosp Psychiatry. 2009 Jul-Aug;31(4):306-15. doi: 10.1016/j.genhosppsych.2009.04.002. Epub 2009 May 13. PMID: 19555789. | Review on physical activity including only studies published until Jan 15th 2018 |
| Sabe M, Sentissi O, Kaiser S. Mind-body therapies for negative symptoms of schizophrenia: Systematic review of randomized controlled trials and meta-analysis. Swiss Archives of Neurology, Psychiatry and Psychotherapy. 2019; 170(Supplement 8),p.39 | Wrong type of review, e.g. meta-review, narrative review |
| Şakir GICA, Selvi Y. Sleep Interventions in the Treatment of Schizophrenia and Bipolar Disorder. Archives of Neuropsychiatry, 58(Suppl 1), S53 (2021). | Wrong type of review, e.g. meta-review, narrative review |
| Scott AJ, Webb TL, Martyn-St James M, Rowse G, Weich S. Improving sleep quality leads to better mental health: A meta-analysis of randomised controlled trials. Sleep Med Rev. 2021 Dec;60:101556. doi: 10.1016/j.smrv.2021.101556. Epub 2021 Sep 23. PMID: 34607184; PMCID: PMC8651630. | Diagnoses other than predefined SMI and/or no subgroup analyses available |
| Siantz E, Aranda MP. Chronic disease self-management interventions for adults with serious mental illness: a systematic review of the literature. Gen Hosp Psychiatry . 2014 May-Jun;36(3):233-44. DOI: 10.1016/j.genhosppsych.2014.01.014. Epub 2014 Feb 10. PMID: 24630896. | Interventions other than predefined lifestyle interventions |
| Stanley SH. Improving the physical health of the mentally ill: recent advances. Current Opinion in Psychiatry 33(5):p 451-459, September 2020. \| DOI: 10.1097/YCO.0000000000000627 | Diagnoses other than predefined SMI and/or no subgroup analyses available |
| Stubbs B, Vancampfort D, Hallgren M, et al. EPA guidance on physical activity as a treatment for severe mental illness: A meta-review of the evidence and position statement from the European Psychiatric Association (EPA), supported by the International Organization of Physical Therapists in Mental Health (IOPTMH). Eur Psychiatry 2018;54:124–44. doi: 10.1016/j.eurpsy.2018.07.004. | Wrong type of review, e.g. meta-review, narrative review |
| Tavares BB, Moraes H, Deslandes AC, Laks J. Impact of physical exercise on quality of life of older adults with depression or Alzheimer’s disease: a systematic review. Trends Psychiatry Psychother. 2014;36(3):134–139 | Review on physical activity including only studies published until Jan 15th 2018 |
| Trkulja V, Barić H. Current Research on Complementary and Alternative Medicine (CAM) in the Treatment of Major Depressive Disorder: An Evidence-Based Review. In: Kim, YK. (eds) Major Depressive Disorder. Advances in Experimental Medicine and Biology, vol 1305. Springer, Singapore (2021). https://doi.org/10.1007/978-981-33-6044-0_20 | Wrong type of review, e.g. meta-review, narrative review |
| Vancampfort D, Probst M, Skjaerven LH, Catalan-Matamoros D. Systematic Review of the Benefits ofPhysical Therapy Within aMultidisciplinary Care Approach for People With Schizophrenia. Phys Ther. 2012 Jan;92(1):11-23. doi: 10.2522/ptj.20110218. Epub 2011 Nov 3. PMID: 22052946. | Review on physical activity including only studies published until Jan 15th 2018 |
| Vancampfort D, Stubbs B, Van Damme T, Smith L, Hallgren M, Schuch F, Deenik J, Rosenbaum S, Ashdown-Franks G, Mugisha J, Firth J. The efficacy of meditation-based mind-body interventions for mental disorders: A meta-review of 17 meta-analyses of randomized controlled trials. J Psychiatr Res. 2021 Feb;134:181-191. doi: 10.1016/j.jpsychires.2020.12.048. Epub 2020 Dec 21. PMID: 33388701. | Wrong type of review, e.g. meta-review, narrative review |
| van Hasselt FM, Krabbe PF, van Ittersum DG, Postma MJ, Loonen AJ. Evaluating interventions to improve somatic health in severe mental illness: a systematic review. Acta Psychiatr Scand. 2013 Oct;128(4):251-60. doi: 10.1111/acps.12096. Epub 2013 Feb 26. PMID: 23438505. | Diagnoses other than predefined SMI and/or no subgroup analyses available |
| Vollbehr NK, Bartels-Velthu isAA, Nauta MH, Castelein S, Steenhuis LA, Hoenders HJR et al. Hatha yoga foracute, chronic and/or treatment-resistant mood and anxiety disorders: A systematic review andmeta-ana lysis. PLoS ONE 13(10): e0204925 (2018). https://doi.org/10.1371/journal. pone.0204925 | Diagnoses other than predefined SMI and/or no subgroup analyses available |
| Warburton DER, Charlesworth S, Ivey A, Nettlefold L, Bredin S: A systematic review of the evidence for Canada’s physical activity guidelines for adults. IJBNPA. 2010, 7 (39): 1-220. | Wrong type of review, e.g. meta-review, narrative review |
| Whale R, Adams C, Bucur M. What are wellbeing treatments for psychosis and where are they hiding? Early Intervention in Psychiatry, 12(Supplement 1), p. 197 (2018). | Wrong type of review, e.g. meta-review, narrative review |
| Wie GX, Yang L, Imm K, Loprinzi PD, Smith L, Zhang X et al. Effects of Mind-Body Exercises on Schizophrenia: A Systematic Review With Meta-Analysis. Front Psychiatry. 2020; 11: 819. 2020 Aug 13. doi: 10.3389/fpsyt.2020.00819. PMCID: PMC7457019. PMID: 32922321. | Review on physical activity including only studies published until Jan 15th 2018 |
| Wang X, Li Y, Fan H. The associations between screen time-based sedentary behavior and depression: a systematic review and meta-analysis. BMC Public Health. 2019 Nov 14;19(1):1524. doi: 10.1186/s12889-019-7904-9. PMID: 31727052; PMCID: PMC6857327. | Wrong type of review, e.g. meta-review, narrative review |
| Yang GY, Wang LQ, Ren J, Zhang Y, Li ML, Zhu YT, Luo J, Cheng YJ, Li WY, Wayne PM, Liu JP. Evidence base of clinical studies on Tai Chi: a bibliometric analysis. PLoS One. 2015 Mar 16;10(3):e0120655. doi: 10.1371/journal.pone.0120655. PMID: 25775125; PMCID: PMC4361587. | Review on physical activity including only studies published until Jan 15th 2018 |
| Yang Z, Xiong GL. Managing Obesity in Patients with Mental Illness: A Literature Review and Implication for Clinical Practice. Current Psychiatry Reviews, 2015, 11, 290-297 (2015). DOI: 10.2174/1573400511666150831201450. | Wrong type of review, e.g. meta-review, narrative review |
| Yang Y, Shin JC, Li D, An R. Sedentary Behavior and Sleep Problems: a Systematic Review and Meta-Analysis. Int J Behav Med. 2017 Aug;24(4):481-492. doi: 10.1007/s12529-016-9609-0. PMID: 27830446. | Interventions other than predefined lifestyle interventions |
| Young CL, Trapani K, Dawson S, O'Neil A, Kay-Lambkin F, Berk M, Jacka FN. Efficacy of online lifestyle interventions targeting lifestyle behaviour change in depressed populations: A systematic review. Aust N Z J Psychiatry. 2018 Sep;52(9):834-846. doi: 10.1177/0004867418788659. Epub 2018 Jul 27. PMID: 30052063. | Outcome measures other than psychopathology, cognitive functioning, quality of life or changes in physical health indicators |
| Zhai L, Zhang Y, Zhang D. Sedentary behaviour and the risk of depression: a meta-analysis. Br J Sports Med. 2015 Jun;49(11):705-9. doi: 10.1136/bjsports-2014-093613. Epub 2014 Sep 2. PMID: 25183627. | Wrong type of review, e.g. meta-review, narrative review |
| Zhang A, Franklin C, Currin-McCulloch J, Park S, Kim J. The effectiveness of strength-based, solution-focused brief therapy in medical settings: a systematic review and meta-analysis of randomized controlled trials. J Behav Med. 2018 Apr;41(2):139-151. doi: 10.1007/s10865-017-9888-1. Epub 2017 Oct 3. PMID: 28975531. | Review on physical activity including only studies published until Jan 15th 2018 |

**S4: Included reviews on multimodal lifestyle interventions**

| Reference | Included studies / n | Popu-lation | Interventions | Control group | Main results | SIGN checklist |
| --- | --- | --- | --- | --- | --- | --- |
| Alvarez-Jimenez M, Hetrick SE, Gonzalez-Blanch C, Gleeson JF, McGorry PD. Non-pharmacological management of antipsychotic-induced weight gain: systematic review and meta-analysis of randomised controlled trials. The British Journal of Psychiatry. 2008;193(2):101-7. | 10 studies relevant for this review  (n = 482) | SCZ | Interventions aimed at preventing or controlling antipsychotic-induced weight gain (including cognitive-behavioural interventions, nutritional counselling interventions, and/ or exercise interventions). | Either standard care or an active comparator intervention, e.g., non-structured information on weight gain or informative group sessions | Compared to TAU, non-pharmacological interventions, cognitive-behavioural therapy, and nutritional counselling, implemented in both group and individual settings, helped reducing weight gain. The effects remained over follow-up. Results regarding the effect on quality of life were scarce and ambiguous. | High quality (++) |
|  |  |  |  |  |  |  |
| Aucoin M, LaChance L, Clouthier SN, Cooley K. Dietary modification in the treatment of schizophrenia spectrum disorders: A systematic review. World journal of psychiatry. 2020;10(8):187.  Update from:  Aucoin M, LaChance L, Cooley K, Kidd S. Diet and Psychosis: A Scoping Review. Neuropsychobiology. 2020;79(1):20-42. | 20 studies relevant for this review  (n = 3017) | SMI (majority SCZ) | Multimodal interventions that attempted to modify participants’ overall dietary pattern such as diet counselling or nutrition education, plus physical exercise and/or psychosocial interventions. | TAU, active control group, waiting list, none | All studies examined multimodal interventions as opposed to solely focusing on diet. The majority of experimental trials reported improvements in at least one mental health domain such as psychotic symptoms, cognition, functioning or quality of life. Studies yielding positive results were more likely to include only patients with schizophrenia spectrum disorder (instead of SMI in general) and to have a group setting. About half of the studies assessing physical health outcomes reported positive results.  There was a high level of heterogeneity regarding patient population, intervention, and study design. The nutrition advice implemented was in general poorly described and compliance was not assessed. | Acceptable (+) |
|  |  |  |  |  |  |  |
| Bauer IE, Gálvez JF, Hamilton JE, Balanzá-Martínez V, Zunta-Soares GB, Soares JC, et al. Lifestyle interventions targeting dietary habits and exercise in bipolar disorder: A systematic review. Journal of psychiatric research. 2016;74:1-7. | 6 studies (n = 296) | BD | Multimodal lifestyle interventions (targeting nutrition, weight loss motivation, physical activity, wellness), a self-management intervention "Life Goals Collaborative Care", integrated risk reduction intervention (IRRI) and cognitive-behavioural therapy | Not specified | Only few studies have focused on lifestyle interventions in people with bipolar disorder. The included studies provide preliminary evidence that multimodal lifestyle interventions are feasible and efficacious in individuals with bipolar disorder. All but one study considered weight or BMI as primary outcome. variable. Other factors such psychological variables and additional physical health parameters should be considered in the future and use questionnaires that reliably assess dietary habits and physical activity. | Acceptable (+) |
|  |  |  |  |  |  |  |
| Bradshaw T, Lovell K, Harris N. Healthy living interventions and schizophrenia: a systematic review. J Adv Nurs. 2005;49(6):634-54. | 6 studies relevant for this review  (n = 48) | SCZ | Interventions to promote healthier lifestyles and/or reduce risk factors for common medical problems such as weight management including exercise, and nutritional education. | Not specified | Weight management studies showed positive outcomes. The quality of the studies, however, was generally poor. Future studies should use larger numbers of participants who are randomly allocated to treatment groups. Results for nutritional education were inconclusive. | Unacceptable (0) |
|  |  |  |  |  |  |  |
| Brown C, Geiszler LC, Lewis KJ, Arbesman M. Effectiveness of Interventions for Weight Loss for People With Serious Mental Illness: A Systematic Review and Meta-Analysis. Am J Occup Ther. 2018;72(5):7205190030p1-p9. | 17 studies (n = 1874) | SMI | Community based weight loss interventions including a range of components such as exercise, educational sessions, motivational interviewing, mentor programs, behaviour therapy, and diet changes. The interventions typically focused on changing a few key behaviours (e.g., eating at least five servings of fruits and vegetables, decreasing sugary drinks), although the targets varied across the interventions. | Not specified | The various community-based lifestyle-focused interventions had a significant effect on weight loss with a small to medium effect size. | Unacceptable (0) |
|  |  |  |  |  |  |  |
| Bruins J, Jörg F, Bruggeman R, Slooff C, Corpeleijn E, Pijnenborg M. The effects of lifestyle interventions on (long-term) weight management, cardiometabolic risk and depressive symptoms in people with psychotic disorders: a meta-analysis. PLoS One. 2014;9(12):e112276. | 25 studies (n = 1518) | SMI | Interventions either targeting overweight patients in order to help them lose weight, or patients in the early stages of their illness in order to help them prevent antipsychotic induced weight gain. Interventions were considered lifestyle interventions when they had a nutritional element, physical activity and/or a psychological intervention aimed at weight loss or weight gain prevention. | Not specified | Lifestyle interventions were effective in both weight loss and weight-gain-prevention with significant long-term effects, two to six months post-intervention. Lifestyle interventions led to significant improvements in waist circumference, triglycerides, fasting glucose, and insulin. No significant effects were found for blood pressure and cholesterol levels. Four studies reported on depressive symptoms and showed a significant effect. | Acceptable (+) |
|  |  |  |  |  |  |  |
| Caemmerer J, Correll CU, Maayan L. Acute and maintenance effects of non-pharmacologic interventions for antipsychotic associated weight gain and metabolic abnormalities: a meta-analytic comparison of randomized controlled trials. Schizophrenia research. 2012;140(1-3):159-68. | 17 studies (n =810) | SMI | Non-pharmacological interventions aimed at preventing or reducing antipsychotic associated weight gain. Treatment involved cognitive behavioural elements (psychoeducation, self-monitoring, teaching behavioural change strategies and/or cognitive restructuring), and nutritional (dietician and nutritionist consultations) and/or exercise interventions (supervised exercise programs). | Not specified | Non-pharmacological interventions led to a significant reduction in weight and body mass index (BMI) when compared with control groups. Intervention patients experienced also significant decreases in waist circumference, percent body fat, glucose levels, insulin, total cholesterol, low density-lipoprotein-cholesterol and triglycerides. Subgroup analyses showed a superiority of non- pharmacological interventions irrespective of treatment duration, individual or group, cognitive behavioural or nutritional interventions, or prevention versus intervention trials. However, weight and BMI were significantly improved only in outpatient trials, but not in inpatient or mixed samples. | Unacceptable (0) |
|  |  |  |  |  |  |  |
| Cimo A, Stergiopoulos E, Cheng C, Bonato S, Dewa CS. Effective lifestyle interventions to improve type II diabetes self-management for those with schizophrenia or schizoaffective disorder: a systematic review. BMC psychiatry. 2012;12:24. | 4 studies (n = 404) | SCZ | The intervention target lifestyle factors associated with diabetes self-care, such as problem-solving skills, education classes, nutrition education or exercise promotion. | Not specified | Diabetes education is effective in improving weight, BMI, and blood glucose levels when it incorporates diet and exercise components, while challenges such as cognition, motivation, and weight gain that may result from antipsychotics should be addressed. | High quality (++) |
|  |  |  |  |  |  |  |
| Coles A, Maksyutynska K, Knezevic D, Agarwal SM, Strudwick G, Dunbar JA, et al. Peer-facilitated interventions for improving the physical health of people with schizophrenia spectrum disorders: systematic review and meta-analysis. Med J Aust. 2022;217 Suppl 7:S22-s8. | 13 studies (n = 2099) | SCZ | Peer-facilitated interventions (peers involved as sole or co-leaders) targeting physical health outcomes | Any randomized control group | A significant pooled intervention effect was found for physical activity and capacity, but not on healthy eating and metabolic measures. Eight of thirteen studies found statistically significant improvements in at least one outcome. The overall certainty of evidence was low. | Low quality  (-) |
|  |  |  |  |  |  |  |
| De Rosa C, Sampogna G, Luciano M, Del Vecchio V, Pocai B, Borriello G, et al. Improving physical health of patients with severe mental disorders: a critical review of lifestyle psychosocial interventions. Expert review of neurotherapeutics. 2017;17(7):667-81. | 19 studies (n = 2647) | SMI | Psychosocial interventions aimed to promote changes in lifestyle, including an informative component on healthy nutrition, physical activity sessions and/or a psychological intervention aimed at weight loss or weight gain prevention. | Not specified | In general, results were heterogeneous. While some studies reported positive effects of the experimental interventions in modifying patients’ lifestyle behaviours and physical health, many other studies did not find any significant improvement compared to TAU or to other non-specific approaches. Interventions differed regarding their format, setting, professional supervision, regarding their active components and their treatment. | Acceptable (+) |
|  |  |  |  |  |  |  |
| Faulkner G, Soundy AA, Lloyd K. Schizophrenia and weight management: a systematic review of interventions to control weight. Acta psychiatrica Scandinavica. 2003;108(5):324-32. | 8 studies relevant for this review  (n = 142) | SCZ | Among others: behavioural/dietary interventions to moderate antipsychotic-related weight gain | Not specified | All behavioural interventions including exercise and nutritional interventions reported small reductions in, or maintenance of, weight. | Acceptable (+) |
|  |  |  |  |  |  |  |
| Faulkner G, Cohn T, Remington G. Interventions to reduce weight gain in schizophrenia. The Cochrane database of systematic reviews. 2007(1):Cd005148. | 5 studies relevant for this review  (n = 258) | SCZ | Weight loss (treatment) and weight maintenance (prevention) studies evaluating (among others) nonpharmacologic adjunctive interventions were included. Typically, interventions incorporate dietary and/or exercise components. Additionally, some studies may include cognitive/behavioural components. | TAU | Conclusion: Modest weight loss can be achieved with non-pharmacological interventions. However, interpretation is limited by the small number of studies, small sample size, short study duration and by variability of the interventions themselves, their intensity and duration. | High quality (++) |
|  |  |  |  |  |  |  |
| Fernández-Abascal B, Suárez-Pinilla P, Cobo-Corrales C, Crespo-Facorro B, Suárez-Pinilla M. In- and outpatient lifestyle interventions on diet and exercise and their effect on physical and psychological health: a systematic review and meta-analysis of randomised controlled trials in patients with schizophrenia spectrum disorders and first episode of psychosis. Neuroscience and biobehavioral reviews. 2021;125:535-68. | 60 studies (n = 4172) | SCZ | Non-pharmacological protocolized interventions based on exercise and/or psychotherapy focusing on changes in diet and physical activity, aiming to promote physical health, psychological wellbeing and/or changes in lifestyle | TAU or a non-protocolized non-active intervention (e.g., casual counselling). RCTs that compared two different active interventions were not eligible (e.g., running and yoga). | An improvement in anthropometric measurements (BMI, weight, waist circumference) was observed post-intervention, persisting after follow-up. Post-intervention benefit was found also for psychotic symptoms severity (also persisting after follow-up), many cognitive domains and physical and global functioning and quality of life. Conversely, no effect was observed in relation to most blood metabolites, blood pressure and non-psychotic psychopathology and spontaneous physical activity. Improvement was generally larger for interventions including exercise, especially moderate/vigorous aerobic exercise, but follow-up maintenance was greater for psychotherapy interventions. | High quality (++) |
|  |  |  |  |  |  |  |
| Gierisch JM, Nieuwsma JA, Bradford DW, Wilder CM, Mann-Wrobel MC, McBroom AJ, et al. Pharmacologic and behavioral interventions to improve cardiovascular risk factors in adults with serious mental illness: a systematic review and meta-analysis. The Journal of clinical psychiatry. 2014;75(5):e424-40. | 11 studies relevant for this review  (n = 799) | SMI | Interventions included among others behavioural interventions targeting weight control, glucose levels, lipid levels, or overall CVD risk. | Not specified | Compared with control groups, weight control was improved with behavioural interventions. However, no significant effects on HbA1c or lipid control were found. | Acceptable (+) |
|  |  |  |  |  |  |  |
| Gliddon E, Barnes SJ, Murray G, Michalak EE. Online and mobile technologies for self-management in bipolar disorder: A systematic review. Psychiatric rehabilitation journal. 2017;40(3):309-19. | 6 studies relevant for the present review  (n = 719) | BD | eHealth and mHealth interventions | Not specified | Only a subset of studies covered approaches to maintain a healthy lifestyle. In general, eHealth programs appear to provide more comprehensive coverage of self-management strategies compared with mHealth programs | Low quality (-) |
|  |  |  |  |  |  |  |
| Happell B, Davies C, Scott D. Health behaviour interventions to improve physical health in individuals diagnosed with a mental illness: A systematic review. International journal of mental health nursing. 2012;21(3):236-47. | 19 studies relevant for this review  (n = 2396) | SMI | Interventions included psychosocial interventions, behaviour change instructions focusing on healthy lifestyle, physical activity and dietary counselling. | Not specified | The majority of studies (16/19) reported improvements in weight and BMI. | Low quality (-) |
|  |  |  |  |  |  |  |
| Hjorth P, Davidsen AS, Kilian R, Skrubbeltrang C. A systematic review of controlled interventions to reduce overweight and obesity in people with schizophrenia. Acta psychiatrica Scandinavica. 2014;130(4):279-89. | 23 studies (n = 707) | SCZ | Non- pharmacological interventions aimed at weight reduction and/or reducing physical illness (diet, exercise and cognitive behavioural therapy, or mixed combinations of the three). | TAU | Interventions showed efficacy in reducing weight and improving physical health parameters. Only one of 23 studies did not result in any physical health improvement. Since no specific intervention can be pointed out as the most suitable, interventions should be implemented in a way suitable for the participants´ daily practice, and as part of standard care offered in the local psychiatric settings. The validity of the review findings is influenced by the lack of studies with high evidence owing to short study periods and small sample sizes. | Low quality (-) |
|  |  |  |  |  |  |  |
| Lee C, Piernas C, Stewart C, Michalopoulou M, Hajzadeh A, Edwards R, et al. Identifying effective characteristics of behavioral weight management interventions for people with serious mental illness: A systematic review with a qualitative comparative analysis. Obes Rev. 2022;23(1):e13355. | 20 studies (n = 515) | SMI | Any behavioural intervention that aimed to support weight management through diet alone or diet and physical activity. | Not specified. | The active interventions resulted in more weight loss when compared with controls. Regular contact, tools to support enactment, and tailored materials were associated with effectiveness. As these are all supplementary strategies, it may be possible to augment behavioural weight management interventions available for the general population to engage people with SMI. | High quality (++) |
|  |  |  |  |  |  |  |
| Loh C, Meyer JM, Leckband SG. A comprehensive review of behavioral interventions for weight management in schizophrenia. Annals of Clinical Psychiatry. 2006;18(1):23-31. | 23 studies (n = 701) | SCZ | Behavioural interventions with the goal of weight loss consisting of behavioural modification techniques, caloric restriction, and psychoeducation. | Not specified | Weight loss was reported in the majority of studies, while the remaining studies showed either maintenance of baseline weight or minimal weight gain. Study participants in the control groups invariably gained weight over time. High drop-out rates, and the absence of extended post-treatment follow-up still limit the conclusions regarding general efficacy of behavioural treatment of obesity in patients with schizophrenia. | Unacceptable (0) |
|  |  |  |  |  |  |  |
| Mazoruk S, Meyrick J, Taousi Z, Huxley A. The effectiveness of health behavior change interventions in managing physical health in people with a psychotic illness: A systematic review. Perspect Psychiatr Care. 2020;56(1):121-40. | 17 studies relevant for this review  (n = 3183) | SCZ | Health behaviour change interventions, defined as aiming to improve physical health through change in health behaviours, such as (among others) diet or exercise. The majority of studies utilized a mixture of psychoeducation, health behaviour change advice, and motivational techniques (i.e., healthy lifestyle education, exercise programs, and goal setting). | Not specified | Health behaviour change interventions can be effective in improving health and psychiatric outcomes in people with psychotic illness. The majority of studies utilized a combination of healthy lifestyle education, motivational techniques, and exercise. There was moderate to strong evidence of overall effectiveness of weight‐management interventions with the strongest evidence on weight loss, followed by BMI reduction and decrease in waist circumference. The studies focusing on exercise interventions report strong evidence of effectiveness of such interventions on exercise behaviour and improved fitness. In addition, there is moderate to strong evidence of effectiveness of this type of intervention on clinical symptoms. | High quality (++) |
|  |  |  |  |  |  |  |
| McKeon G, Papadopoulos E, Firth J, Joshi R, Teasdale S, Newby J, et al. Social media interventions targeting exercise and diet behaviours in people with noncommunicable diseases (NCDs): A systematic review. Internet Interv. 2022;27:100497. | 2 studies relevant for this review  (n = 45) | SMI | Online social media interventions targeting physical activity and/or diet (as part of an intervention with other modes of delivery). Social media was defined as web-based applications that allow individuals to interact in a virtual community by exchanging user-generated information (e.g., face-to-face lifestyle intervention, online discussion boards, online bulletin boards, chat rooms, online community). | Usual care or no-intervention controls were included. Studies without a control group were eligible for inclusion if they reported pre and post intervention data. | Studies in people with SMI show feasibility and high engagement despite some discrepancy regarding participant satisfaction. Improvements in fitness were non-significant, maybe due to an underpowered design. | High quality (++) |
|  |  |  |  |  |  |  |
| Meader N, Melton HA, Evans C, Wright K, Shiers D, Ratschen E, et al. Multiple versus single risk behaviour interventions for people with severe mental illness: a network meta-analysis and qualitative synthesis. Health and Social Care Delivery Research. 2022. | 47 studies relevant for this review, exact n not given | SMI | Interventions targeting (among others) at least one of the health risk behaviours physical inactivity or unhealthy diet. | TAU or TAU and active control | Interventions targeting physical activity, diet, or a combination of both lead to positive, but modest improvement of the health outcomes weight and BMI, with only small differences in effect. | Acceptable (+) |
|  |  |  |  |  |  |  |
| Mohanty K, Gandhi S, Krishna Prasad M, John AP, Bhaskarapillai B, Malo P, et al. Effectiveness of lifestyle intervention on prevention/management of antipsychotic-induced weight gain among persons with severe mental illness: A systematic review and meta-analysis. J Health Psychol. 2024:13591053241227384. | 12 studies  (n = 2681) | SMI | Weight management interventions including physical activity, diet, counselling and/or motivational interviewing. Duration varied between 6 weeks and 2 years | TAU | The meta-analysis showed no significant effects of interventions on waist circumference, weight or BMI, although a trend towards reduction could be observed for all three. | Low quality  (-) |
|  |  |  |  |  |  |  |
| Mucheru D, Hanlon MC, McEvoy M, Thakkinstian A, MacDonald-Wicks L. Comparative efficacy of lifestyle intervention strategies targeting weight outcomes in people with psychosis: a systematic review and network meta-analysis. JBI Database System Rev Implement Rep. 2019;17(9):1770-825. | 32 studies (n = 3671) | SCZ | Studies designed to deliver nonpharmacological lifestyle interventions such as weight management, diet, or exercise interventions. Most lifestyle intervention studies contained both a dietary and physical activity component. | Not specified. | Results showed that studies with a structured approach for both diet and physical activity demonstrated significant decreases in weight and body mass index in people with psychosis leading to decreases in weight of about 4 kg and reductions in BMI by about 2.5 points. Waist circumference subgroup comparisons mainly comprised single studies and findings were inconclusive. The main cognitive behavioural / motivational strategies utilized in these studies were education, personalized plans or goals, and progress review. In general, the findings were weakened by an increased risk of bias, complex and multicomponent study designs, and lack of clarity in reporting of study methodology. | High quality (++) |
|  |  |  |  |  |  |  |
| Naslund JA, Whiteman KL, McHugo GJ, Aschbrenner KA, Marsch LA, Bartels SJ. Lifestyle interventions for weight loss among overweight and obese adults with serious mental illness: A systematic review and meta-analysis. Gen Hosp Psychiatry. 2017;47:83-102. | 17 studies (n = 1968) | SMI | Intervention involved non-pharmacological lifestyle programs (including diet, nutrition education, exercise, or physical activity) for weight loss targeting overweight and obese adults with serious mental illness. | Not specified | Lifestyle interventions appear to be effective for treating overweight and obesity among people with serious mental illness. Interventions of ≥ 12-months duration compared to ≤ 6-months duration appear to achieve more consistent outcomes, though effect sizes are similar for both shorter and longer duration interventions. | Acceptable (+) |
|  |  |  |  |  |  |  |
| Nover C, Jackson SS. Primary care-based educational interventions to decrease risk factors for metabolic syndrome for adults with major psychotic and/or affective disorders: a systematic review. Syst Rev. 2013;2:116. | 0 studies included | SMI | Non-pharmacological, education-based interventions to address metabolic syndrome risk factors of patients treated in the primary care sector. | Not specified | The authors were unable to identify any studies meeting a priori inclusion criteria because there were no primary care-based studies. | Low quality (-) |
|  |  |  |  |  |  |  |
| Oliveira ACN, Guariente SMM, Zazula R, Mesas AE, Oliveira CEC, Reiche EMV, et al. Hybrid and Remote Psychosocial Interventions Focused on Weight and Sedentary Behavior Management Among Patients with Severe Mental Illnesses: a Systematic Review. Psychiatr Q. 2022;93(3):813-40. | 16 studies (n = 1884) | SMI | Psychosocial interventions to modify behaviour through psychoeducation intervention or digital technologies and aiming at decreasing anthropometric measures (i.e., weight, BMI, waist, or abdominal circumference), and improve health behaviour, such as reduce sedentary behaviour and improve physical activity. Digital technologies could use mobile phone applications, pedometers or regular email, text message, or phone calls contact (summarized as remote interventions) or digital interventions associated with face-to-face strategies (summarized as hybrid interventions). | Not specified | The results show that the use of digital devices and strategies, such as pedometers, phone calls, and social media might be feasible and useful to reduce sedentary behaviour and increase the frequency of physical activity; all remote interventions and six of nine hybrid interventions found significant outcomes in favour of their interventions. They also might be valid to improve general health parameters, such as weight, BMI, abdominal circumference, as well as cardiovascular conditioning. Most interventions used digital pedometers and mobile phone communication (either text messages or phone calls) as main strategies. In both remote and hybrid interventions especially studies with larger sample size found significant effects. | Low quality (-) |
|  |  |  |  |  |  |  |
| Olker SJ, Parrott JS, Swarbrick MA, Spagnolo AB. Weight management interventions in adults with a serious mental illness: A meta-analytic review. American Journal of Psychiatric Rehabilitation. 2016;19(4):370-93. | 14 studies (n = 1779) | SMI | Multicomponent approaches (defined as mixed interventions that promote increased physical activity and improved nutritional intake) to weight management for adults with SMI who live in the community. Weight management programs of interest included three of the following components: nutritional counselling/dietary therapy or physical activity, consistent with cognitive and behavioural theories or the practice of motivational interviewing, and formatted to provide additional support, mentoring, or coaching opportunities from other persons with mental illness and a goal of weight loss or management. | TAU (in most cases considered as regular mental health care plus general health advisement) | Across all trials, there was an effect in favour of the intervention groups, with a mean absolute weight reduction of -2.01 kg compared to controls over a period of 3 to 12 months. For BMI, a mean change of - 1.71 kg/m2 was observed in favour of the intervention group compared to the controls. In contrast to changes in weight and BMI, between-group differences in treatment effects for mean change in waist circumference were not statistically significant, although the direction of the effect was consistent with the weight and BMI results in favour of the intervention groups. Subgroup meta-analyses suggested that programs that include individual sessions and are delivered at the onset of the disease may have the greatest impact on weight management. Despite the statistically significant findings of mean weight change in the intervention groups compared to controls, the reported weight loss is only 2% of baseline body weight. This falls short of the clinically significant target of 5% weight loss to reduce associated health complications. | Acceptable (+) |
|  |  |  |  |  |  |  |
| Pape LM, Adriaanse MC, Kol J, van Straten A, van Meijel B. Patient-reported outcomes of lifestyle interventions in patients with severe mental illness: a systematic review and meta-analysis. BMC psychiatry. 2022;22(1):261. | 36 studies (n = 5907) | SMI | Non-pharmacological lifestyle interventions promoting weight loss, weight management, healthy diet, decrease of sedentary behaviour, or increase of physical activity. | Nonactive or minimally active control conditions (e.g. TAU or waitlist control group) | Lifestyle interventions had no significant effect on quality of life, with high heterogeneity. A small effect on the severity of depression and a moderate effect on the severity of anxiety could be detected. Sixteen trials examined effects on physical activity. Eight of these reported improvements in physical activity in the intervention groups in terms of more minutes of exercise per week, higher vigorous activity scores, and less time spent sitting. Sixteen trials assessed dietary behaviour. Three trials found significant improvements in reducing fat consumption, short-term increases in fruit and vegetable consumption, and adherence to the Mediterranean diet. Three other trials found significant changes in willingness to change dietary behaviour in favour of the intervention. The overall quality of the trials was low, and only seven of the 36 trials had a low risk of bias. | High quality (++) |
|  |  |  |  |  |  |  |
| Simjanoski M, Patel S, Boni R, Balanzá-Martínez V, Frey BN, Minuzzi L, et al. Lifestyle interventions for bipolar disorders: A systematic review and meta-analysis. Neuroscience and biobehavioral reviews. 2023;152:105257. | 4 studies relevant for this review  (n = 192) | BD | Lifestyle interventions (in person and/or digital) on any of the domains diet, physical activity, sleep, social relationships, and/or stress management (among others). | No intervention or any other intervention that is not an active lifestyle intervention or novel medication treatment | Despite the small number of studies included, there were promising results for the efficacy of the interventions covering diet, physical activity and sleep on the reduction of depressive symptoms and the improvement of overall functioning. There was a trend of higher efficacy rates among interventions targeting 3 or 4 domains in comparison to 1 or 2 domains. | High quality (++) |
|  |  |  |  |  |  |  |
| Singh VK, Karmani S, Malo PK, Virupaksha HG, Muralidhar D, Venkatasubramanian G, et al. Impact of lifestyle modification on some components of metabolic syndrome in persons with severe mental disorders: A meta-analysis. Schizophrenia research. 2018;202:17-25. | 19 studies (n = 3213) | SMI | Lifestyle/psychosocial interventions having components of nutrition elements, physical activity, cognitive behavioural treatment, behavioural therapy, or psychoeducation targeting weight change or management to improve metabolic syndrome among persons with severe mental disorders. | TAU | Lifestyle interventions had significantly superior efficacy in the reducing weight, BMI, and waist circumference when compared to TAU. | Low quality (-) |
|  |  |  |  |  |  |  |
| Speyer H, Jakobsen AS, Westergaard C, Nørgaard HCB, Jørgensen KB, Pisinger C, et al. Lifestyle Interventions for Weight Management in People with Serious Mental Illness: A Systematic Review with Meta-Analysis, Trial Sequential Analysis, and Meta-Regression Analysis Exploring the Mediators and Moderators of Treatment Effects. Psychother Psychosom. 2019;88(6):350-62. | 41 studies (n = 4267) | SMI | Individualized lifestyle interventions for weight management aimed at decreasing body weight by modifying energy balance through improved diet, increased physical activity, or both. These approaches may include techniques to modify behaviour, such as psychoeducation, psychological counselling, motivational interviewing, stages of change, or cognitive therapy. | Any control (concurrent control condition or TAU) | There is a statistically significant but clinically insignificant mean effect of individualized lifestyle interventions for weight loss in people with SMI. The experimental interventions reduced the mean difference in BMI by -0.63 kg/m2 compared to the control groups. At post-intervention follow-up, the effect size remained similar but was no longer significant. The risk ratio for losing ≥5% of baseline weight was 1.51 compared to the control groups. The experimental interventions also reduced waist circumference by 2.1 cm compared to the control group. Quality of life was not statistically different between the control and intervention groups. Individual sessions were more effective than group sessions. The quality of the evidence was low or very low. | High quality (++) |
|  |  |  |  |  |  |  |
| Stevens H, Smith J, Bussey L, Innerd A, McGeechan G, Fishburn S, et al. Weight management interventions for adults living with overweight or obesity and severe mental illness: a systematic review and meta-analysis. Br J Nutr. 2023;130(3):536-52. | 14 studies relevant for this review  (n = 1046) | SMI | Weight management interventions aimed at people living with SMI and overweight or obesity, including physical activity, diet, psychological interventions, and group education. | TAU, no care or any other weight management intervention | There was a small effect of interventions on body weight according to a meta-analysis of three randomized controlled trials, which was not observed in three quasi-experimental studies. Meta-analysis of four randomized controlled trials showed no effect of interventions on BMI, while only one of four quasi-experimental studies showed a large effect on BMI.  Results on quality of life outcomes were mixed, while no effect on mental health was observed.  Future studies should take mobility and physical health challenges of people with SMI into account and incorporate qualitative methodology in order to gain insight into potential weight management barriers and facilitators. | High quality  (++) |
|  |  |  |  |  |  |  |
| Stubbs B, Williams J, Shannon J, Gaughran F, Craig T. Peer support interventions seeking to improve physical health and lifestyle behaviours among people with serious mental illness: A systematic review. International journal of mental health nursing. 2016;25(6):484-95. | 7 studies (n = 220) | SMI | Peer support interventions addressing any physical health outcome or lifestyle factors (including physical activity, nutrition). Peer support was defined as involving one or more persons who have a history of mental illness and who have experienced significant improvements in their psychiatric condition offering services and/or supports to other people with SMI. | Not specified | There is inconsistent evidence on the use of peer support workers for physical health improvement and lifestyle change in people with SMI. Small sample sizes, heterogeneity of interventions and outcome measures, and lack of clarity about the unique contribution of peer support workers prevent definitive conclusions about the benefits of peer support workers and physical health in SMI. | Acceptable (+) |
|  |  |  |  |  |  |  |
| Taylor J, Stubbs B, Hewitt C, Ajjan RA, Alderson SL, Gilbody S, et al. The Effectiveness of Pharmacological and Non-Pharmacological Interventions for Improving Glycaemic Control in Adults with Severe Mental Illness: A Systematic Review and Meta-Analysis. PLoS One. 2017;12(1):e0168549. | 13 studies relevant for this review  (n = 1302) | SMI | (Among others) behavioural non-pharmacological lifestyle interventions such as weight loss programs and/or physical exercise programs combined with educational and behavioural strategies promoting a healthier lifestyle. | Any control group | Behavioural interventions were effective in reducing fasting glucose, but not HbA1c, compared with usual care. Behavioural interventions of longer duration and those that included repeated physical activity had greater effects on fasting glucose than those without these characteristics. Baseline levels of fasting glucose explained some of the heterogeneity in behavioural interventions. Interventions lasting more than 6 months had an even greater effect. | High quality (++) |
|  |  |  |  |  |  |  |
| Tosh G, Clifton AV, Xia J, White MM. General physical health advice for people with serious mental illness. The Cochrane database of systematic reviews. 2014(3):Cd008567.  Update from: Tosh G, Clifton A, Bachner M. General physical health advice for people with serious mental illness. The Cochrane database of systematic reviews. 2011(2):Cd008567. | 7 studies (n = 1113) | SMI | General physical health advice, defined as preventative information or counsel having an educative component, a preventative aim, and an ethos of self-empowerment. | Any control group | There is some limited evidence that the provision of physical healthcare advice can improve mental but not physical aspects of health-related quality of life. | High quality (++) |
|  |  |  |  |  |  |  |
| Tumiel E, Wichniak A, Jarema M, Lew-Starowicz M. Nonpharmacological interventions for the treatment of cardiometabolic risk factors in people with schizophrenia—a systematic review. Frontiers in psychiatry. 2019;10:566. | 20 studies (n = 1153) | SCZ | Physical activity (e.g., HIT, walks, aerobic training, resistance training), diet, and psychotherapy (motivational and educational interventions) | Not specified | Almost all interventions appeared to have benefits, either towards improving metabolic parameters, cardiovascular fitness, or health perception. Results show that healthy diet educational intervention, as well as physical exercise, could reduce weight in patients with schizophrenia. Additional educational and motivational interventions did not seem to further improve results compared to programs involving only physical activity. Many studies were found to have low methodological quality, involving single-group pre–post, uncontrolled feasibility, or quasi-experimental designs. | Low quality (-) |
|  |  |  |  |  |  |  |
| Veit T, Barnas C. Diet blues: Methodological problems in comparing non-pharmacological weight management programs for patients with schizophrenia. International journal of psychiatry in clinical practice. 2009;13(3):173-83. | 39 studies; (n = 2765) | SCZ | Weight management programs | Not specified | Apart from one single intervention all studies showed either a reduction or at least maintenance of weight. In general, behavioural therapy is understudied and there are relatively few controlled trials. | Unacceptable (0) |
|  |  |  |  |  |  |  |
| Verhaeghe N, De Maeseneer J, Maes L, Van Heeringen C, Annemans L. Effectiveness and cost-effectiveness of lifestyle interventions on physical activity and eating habits in persons with severe mental disorders: a systematic review. The international journal of behavioral nutrition and physical activity. 2011;8:28. | 14 studies (n = 669) | SMI | Psychoeducational and/or behavioural interventions on physical activity and/or eating habits | Not specified | Weight loss and Body Mass Index decrease were observed in intervention groups in 11 of 14 studies. The difference in weight change between intervention and control groups was statistically significant in nine studies. In general, reductions in body weight of 5.0% or more are considered to greatly reduce the risks of physical health problems. In the included trials, no study achieved this target. Differences in mean Body Mass Index between intervention and control groups were statistically significant in eight studies. Five studies reported improvements in quality of life and general health. In none of the studies cost-effectiveness of lifestyle interventions was examined. | Low quality (-) |
|  |  |  |  |  |  |  |
| Whitney Z, Procyshyn RM, Fredrikson DH, Barr AM. Treatment of clozapine-associated weight gain: a systematic review. European journal of clinical pharmacology. 2015;71(4):389-401. | 1 study relevant for this review  (n = 53) | SCZ | Among others non-pharmacological interventions to attenuate or reverse clozapine-associated weight gain (diet and physical activity program) | Not specified. | Behavioural and nutritional interventions show modest effects on decreasing clozapine-associated weight gain. | Low quality (-) |
|  |  |  |  |  |  |  |
| Zavala GA, Todowede O, Mazumdar P, Aslam F, Choudhury AH, Jarde A, et al. Effectiveness of interventions to address obesity and health risk behaviours among people with severe mental illness in low- and middle-income countries (LMICs): a systematic review and meta analysis. Glob Ment Health (Camb). 2022;9:264-73. | 4 studies relevant for this review  (n = 283) | SCZ | (Among others) any non-pharmacological intervention targeting weight reduction and health risk behaviour in people with SMI of low-and middle-income countries | TAU, no intervention, placebo, very brief intervention, usual care | Lifestyle interventions to reduce weight in people with SMI were effective. The quality and sample size of the studies was not optimal, most were small studies, with inadequate power to evaluate the primary outcome. | High quality (++) |

Note: BD: bipolar disorder, MD: major depression, SCZ: schizophrenia, SMI: severe mental illnesses, TAU: treatment as usual

**S5: Included reviews on physical activity**

| Reference | Included studies / n | Popu-lation | Interventions | Control group | Main results | SIGN checklist |
| --- | --- | --- | --- | --- | --- | --- |
| Ashdown-Franks G, Williams J, Vancampfort D, Firth J, Schuch F, Hubbard K, et al. Is it possible for people with severe mental illness to sit less and move more? A systematic review of interventions to increase physical activity or reduce sedentary behaviour. Schizophrenia research. 2018. | 32 studies (n = 2843) | SMI | Physical Activity (light, moderate, vigorous, moderate-vigorous, total, or daily steps) | Controlled trials and uncontrolled pre-and post intervention trials | Of 16 controlled trials, 7 (47%) reported significant improvements in physical activity although only one found changes with an objective measure while of 16 uncontrolled trials, 3 (20%) found improvements in physical activity (one with objective measurement). There is inconsistent and low-quality evidence to show that interventions can be effective in changing physical activity or sedentary behaviour in this population. | Unacceptable (0) |
|  |  |  |  |  |  |  |
| Bredin SS, Kaufman KL, Chow MI, Lang DJ, Wu N, Kim DD, et al. Effects of aerobic, resistance, and combined exercise training on psychiatric symptom severity and related health measures in adults living with schizophrenia: A systematic review and meta-analysis. Frontiers in Cardiovascular Medicine. 2022;8:753117. | 22 studies (n = 913) | SCZ | Aerobic or resistance exercise, or a combination of both aerobic and resistance training for a minimum duration of six weeks or longer. | Not specified | Aerobic training resulted in a significant decrease in Positive and Negative Syndrome Scale (PANSS) negative scores and PANSS general scores. Four studies combined in a meta-analysis found no significant effect of aerobic training on PANSS positive scores. Resistance training did not lead to significant effects on PANSS total scores. Combined aerobic and resistance training had no significant effect on body mass index, PANSS positive scores, or PANSS total scores. However, grouping together the results from all exercise training modalities revealed significant effects on body mass index, maximal/peak oxygen consumption, body weight, and negative symptoms. | High quality (++) |
|  |  |  |  |  |  |  |
| Brinsley J, Schuch F, Lederman O, Girard D, Smout M, Immink MA, et al. Effects of yoga on depressive symptoms in people with mental disorders: a systematic review and meta-analysis. British Journal of Sports Medicine. 2021;55(17):992-1000. | 19 studies (n = 1080) | SMI | Yoga intervention comprising ≥ 50% movement component/ physical activity | Treatment as usual, waitlist or attention controls | Yoga showed greater reductions in depressive symptoms than control groups. Greater reductions in depressive symptoms were associated with higher frequency of yoga sessions per week.  Subgroup analyses based on diagnostic groups revealed evidence for a moderate effect of yoga on depressive symptoms in depressive disorders and a large effect in schizophrenia. | Acceptable (+) |
|  |  |  |  |  |  |  |
| Brupbacher G, Gerger H, Zander-Schellenberg T, Straus D, Porschke H, Gerber M, et al. The effects of exercise on sleep in unipolar depression: a systematic review and network meta-analysis. Sleep medicine reviews. 2021;59:101452. | 7 studies (n = 1645) | MD | Aerobic, strength or meditative movement exercise | At least one other comparator group (no specification, e.g. TAU, active or passive control group). | The network meta-analysis showed that compared to a passive control condition, all exercise interventions except moderate aerobic exercise alone resulted in significantly better sleep outcomes.  Active control, mind-body exercise, treatment as usual, and vigorous aerobic exercise resulted in similar effects. Mind-body combined with treatment as usual and vigorous strength training were the only treatments which were superior when compared to treatment as usual.  The network meta-analysis is statistically very robust with low heterogeneity, incoherence, and indirectness. However, confidence in the findings was moderate to very low, primarily due to within-study bias. | High quality (++) |
|  |  |  |  |  |  |  |
| Cempa K, Jurys T, Kluczyński S, Andreew M. Physical activity as a therapeutic method for non-pharmacological treatment of schizophrenia: A systematic literature review. Psychiatr Pol. 2022;56(4):837-59. | 17 studies  (n = 1206) | SCZ | Any physical activity intervention | TAU, active control (e.g. aerobic exercise, strength training, video games, stretching) | Physical activity interventions yielded mixed results, with some studies observing a positive effect on symptoms of schizophrenia. | Unacceptable  (0) |
|  |  |  |  |  |  |  |
| Chang KT, Goh KK, Latthirun K, Yang CT. The effect of exercise on cognition and clinical symptoms of patients with schizophrenia: A systematic review of randomized controlled trial. Prog Brain Res. 2024;283:255-304. | 27 studies (n = 1459) | SCZ | At least one exercise intervention (also including the combination with cognitive training but no single-session intervention), e.g. aerobic exercise, combined aerobic and resistance training, high intensity interval training, yoga, material arts | Control group or non-exercise intervention group | Exercise interventions showed significant improvements in clinical symptoms, especially negative symptoms, compared to control groups. In addition, exercise interventions enhanced overall functionality (psychological, social, occupational) and the improvement seems to be tied to improvements in physical fitness. Cognitive functioning also seems to improve through exercise, with most pronounced findings for higher-order cognitive functions, including processing speed, attention, and working memory. | High quality (++) |
|  |  |  |  |  |  |  |
| Chen C, Shan W. Pharmacological and non-pharmacological treatments for major depressive disorder in adults: A systematic review and network meta-analysis. Psychiatry research. 2019;281:112595. | 91 studies (n = 10991), exact numbers of exercise trials relevant for this review not given | MD | Among others: Physical exercise | Any inactive intervention and active treatments investigated by the review | Network meta-analysis for efficacy showed that exercise was more effective than control groups measured by response rate. | Acceptable (+) |
|  |  |  |  |  |  |  |
| Gallardo-Gómez D, Noetel M, Álvarez-Barbosa F, Alfonso-Rosa RM, Ramos-Munell J, Del Pozo Cruz B, et al. Exercise to treat psychopathology and other clinical outcomes in schizophrenia: A systematic review and meta-analysis. Eur Psychiatry. 2023;66(1):e40. | 28 studies (n = 1460) | SCZ | Exercise interventions | Non-exercise treatment or another exercise intervention | Supervised exercise significantly improved psychopathology in SCZ. Exercise was consistently superior to the provision of usual treatment and occupational therapy. Positive effects on psychopathology were stronger in outpatients compared to inpatients. Overall, benefits were independent of the type of exercise performed. Exercise also improves muscle strength and self-reported disability. | High quality (++) |
|  |  |  |  |  |  |  |
| Guo L, Kong Z, Zhang Y. Qigong-based therapy for treating adults with major depressive disorder: a meta-analysis of randomized controlled trials. International journal of environmental research and public health. 2019;16(5):826. | 7 studies (n = 382) | MD | Interventions with Qigong as a core intervention | Passive or active control group as a comparison, such as a waitlist, TAU, or walking | The pooled results indicated that qigong-based therapy has a significant benefit on depression severity in individuals with major depressive disorder. Specifically, qigong led to significantly reduced depression as compared to the active and the passive control groups, respectively. For studies which reported categorical outcomes, qigong intervention showed significantly improved treatment response and remission rates in comparison to the waitlist control group. | Unacceptable (0) |
|  |  |  |  |  |  |  |
| Guo J, Liu K, Liao Y, Qin Y, Yue W. Efficacy and feasibility of aerobic exercise interventions as an adjunctive treatment for patients with schizophrenia: a meta-Analysis. Schizophrenia (Heidelb). 2024;10(1):2.. | 17 studies  (n = 973) | SCZ | Aerobic exercise or aerobic exercise plus psychological intervention as adjunctive treatment. Intervention duration ranged from one to twelve months. | TAU | Aerobic exercise significantly reduced symptoms of schizophrenia (as assessed by PANSS) according to a meta-analysis of ten RCTs. Negative symptoms in particular were alleviated.  Subgroup analyses revealed that a treatment duration of two to three months, and an exercise duration of 100 to 220 min per week were most effective.  BMI was not reduced by aerobic exercise. | High quality  (++) |
|  |  |  |  |  |  |  |
| Heissel A, Heinen D, Brokmeier LL, Skarabis N, Kangas M, Vancampfort D, et al. Exercise as medicine for depressive symptoms? A systematic review and meta-analysis with meta-regression. Br J Sports Med. 2023. | 20 studies relevant for this review  (n = 1129) | MD | Aerobic or resistance training, or a mix of both, ranging in duration from 1.5 to 32 weeks. | TAU, health education, placebo, waitlist, social conversation | Exercise had a large beneficial effect on depressive symptoms in people with MD according to a meta-analysis of 20 randomised controlled clinical trails. This effect remained significant (albeit with a smaller effect size) even when the analysis was restricted to 9 low-risk bias studies.  Subgroup analyses showed a large effect for both aerobic and resistance (but not mixed) exercise, for moderate and high (but not low) intensity exercise, for supervised (but not unsupervised) exercise, and for both group and individual exercise.  Moderating variables were identified using meta-regression: Higher antidepressant use in the control group was associated with smaller effects, as were trials with a longer duration in weeks (potentially due to higher drop out).  The Number Needed to Treat for exercise was 1.9, which is comparable to psychotherapy for depression. | High quality  (++) |
|  |  |  |  |  |  |  |
| Kim M, Lee Y, Kang H. Effects of Exercise on Positive Symptoms, Negative Symptoms, and Depression in Patients with Schizophrenia: A Systematic Review and Meta-Analysis. Int J Environ Res Public Health. 2023;20(4). | 15 studies (n = 1642) | SCZ | Any type of exercise (aerobic, resistance, multicomponent, neuromotor) | Usual care, no intervention, stretching | Exercise significantly reduced negative and positive symptoms with a medium and small effect, respectively. No significant effect on depressive symptoms was found. In particular, multicomponent exercise intervention combined with aerobic and resistance exercises and a duration of 20 weeks or more had a moderate effect size in improving positive and negative symptoms. | High quality (++) |
|  |  |  |  |  |  |  |
| Korman N, Armour M, Chapman J, Rosenbaum S, Kisely S, Suetani S, et al. High Intensity Interval training (HIIT) for people with severe mental illness: A systematic review & meta-analysis of intervention studies–considering diverse approaches for mental and physical recovery. Psychiatry research. 2020;284:112601. | 9 studies (n = 366) | SMI | High intensity interval training interventions (HIIT) | Not specified | Following HIIT, there was a moderate improvement in depressive symptoms and cardiorespiratory fitness. HIIT improved depressive symptoms more than moderate intensity continuous training. | Acceptable (+) |
|  |  |  |  |  |  |  |
| Martland R, Korman N, Firth J, Stubbs B. The efficacy of exercise interventions for all types of inpatients across mental health settings: A systematic review and meta-analysis of 47 studies. Journal of sports sciences. 2023;41(3):232-71. | 37 studies relevant for this review  (n = 1306) | SMI | Exercise interventions in an inpatient treatment setting (including aerobic and/or non-aerobic exercise without mindfulness components) | Any control group | Exercise appears to be safe, viable and effective as therapeutic interventions in inpatient mental health settings. Evidence regarding the effects on physical health parameters was inconclusive. The majority of trials reported a reduction in depressive symptoms in MD, an improvement of psychiatric symptoms for SCZ and one retrospective cohort study reported lower symptoms after a walking group intervention in BD. | High quality (++) |
|  |  |  |  |  |  |  |
| Oliva HNP, Monteiro-Junior RS, Oliva IO, Powers AR. Effects of exercise intervention on psychotic symptoms: A meta-analysis and hypothetical model of neurobiological mechanisms. Progress in neuro-psychopharmacology & biological psychiatry. 2023;125:110771. | 16 studies (n = 627) | SCZ | Exercise/physical activity (i.e., aerobic and/or resistance training; individual and group settings) | Not specified (but control group necessary) | Exercise significantly improved schizophrenia symptoms as measured by the Positive and Negative Syndrome Scale (positive, negative, total symptoms; general symptoms after removal of one study in a sensitivity analysis). The effect size was large for negative and general symptoms. | High quality (++) |
|  |  |  |  |  |  |  |
| Pérez Bedoya É A, Puerta-López LF, López Galvis DA, Rojas Jaimes DA, Moreira OC. Physical exercise and major depressive disorder in adults: systematic review and meta-analysis. Sci Rep. 2023;13(1):13223. | 9 studies  (n = 678) | MD | Supervised aerobic and resistance exercise interventions, ranging in duration from 8 to 16 weeks. | Flexibility training, sertraline, cognitive behavioural therapy | According to a meta-analysis of seven randomised controlled clinical trails, there was no significant effect of exercise on depressive symptoms or quality of life compared to control groups. Participants had not previously received antidepressants or psychotherapy. | High quality  (++) |
|  |  |  |  |  |  |  |
| Ren FF, Hillman CH, Wang WG, Li RH, Zhou WS, Liang WM, et al. Effects of aerobic exercise on cognitive function in adults with major depressive disorder: A systematic review and meta-analysis. Int J Clin Health Psychol. 2024;24(2):100447. | 12 studies  (n = 979) | MD | Aerobic exercise interventions or interventions combining aerobic exercise with other interventions, ranging in duration from 3 to 24 weeks. | TAU, placebo medication, active control (e.g. resistance training, art therapy, stretching, relaxation) | A meta-analysis of 12 randomised controlled clinical trials found that aerobic exercise had a small to moderate beneficial effect on overall cognitive function and the subdomains of memory and executive function, but not on attention and processing speed.  Subgroup analyses showed that interventions were more effective when sessions lasted < 45 min (rather than ≥ 45 min), when participants were inpatients (rather than outpatients), and when patients were taking antidepressants. | High quality  (++) |
|  |  |  |  |  |  |  |
| Rißmayer M, Kambeitz J, Javelle F, Lichtenstein TK. Systematic Review and Meta-analysis of Exercise Interventions for Psychotic Disorders: The Impact of Exercise Intensity, Mindfulness Components, and Other Moderators on Symptoms, Functioning, and Cardiometabolic Health. Schizophr Bull. 2024. | 40 studies  (n = 2111) | SCZ | Supervised physical activity interventions (including mind-body exercises if the primary focus was physical activity), ranging in duration from 4 to 32 weeks | TAU, active control (stretching, unsupervised training, video games, table soccer, psychoeducation, occupational therapy) | Physical activity had beneficial small-moderate effects on positive and negative symptoms of schizophrenia and quality of life, large effects on depressive symptoms, but none on BMI, according to meta-analyses of 40 RCTs.  Intervention modalities and components were further analysed with sub-analyses: Moderate-intensity training was more effective than low-intensity in reducing PANSS total score and depressive symptoms. More frequent sessions were more effective in improving psychosocial functioning than less frequent sessions. Interventions including mind-body exercises were more effective in reducing positive symptoms and PANSS general score compared to interventions without mind-body exercises, but were more heterogeneous, potentially indicating higher instructor-dependence.  Depressive symptoms were reduced more effectively in younger than older patients. | High quality  (++) |
|  |  |  |  |  |  |  |
| Roberts MT, Lloyd J, Välimäki M, Ho GW, Freemantle M, Bekefi AZ. Video games for people with schizophrenia. Cochrane Database of Systematic Reviews. 2021(2). | 2 studies relevant for this review  (n = 73) | SCZ | Relevant for the current our review: exergame interventions, in which players use bodily movements to control the game | Control groups consisting of standard care or non-exergames | There is very limited evidence suggesting a possible benefit of exergames compared to standard care in terms of cognitive functioning and aerobic fitness. This finding needs to be replicated in trials with a larger sample size and a longer study duration. | High quality (++) |
|  |  |  |  |  |  |  |
| Sabe M, Sentissi O, Kaiser S. Meditation-based mind-body therapies for negative symptoms of schizophrenia: Systematic review of randomized controlled trials and meta-analysis. Schizophrenia research. 2019;212:15-25. | 12 studies relevant for this review  (n = 862) | SCZ | Yoga, tai chi, qigong, or mindfulness intervention with a duration of intervention of at least 30 min for a total of 8 h of practice over a minimum of 3 weeks | TAU or non-specific control intervention (such as waitlist). | Subgroup analysis yielded no significant effects for the two tai chi studies. For the 10 yoga studies, a significant beneficial effect on negative symptoms score was found but in presence of high heterogeneity. A meta-regression conducted in the yoga study subgroup did not show that total hours of practice predicted severity of negative symptoms at endpoint. Sensitivity analysis including only studies with a low risk of bias showed no beneficial effect for yoga interventions. Subgroup analysis regarding positive symptoms showed a beneficial effect for yoga. However, the small improvement of positive symptoms disappeared after exclusion of an outlier and should thus interpreted with caution. PANSS general symptoms showed a beneficial subgroup effect for yoga. | High quality (++) |
|  |  |  |  |  |  |  |
| Sabe M, Kaiser S, Sentissi O. Physical exercise for negative symptoms of schizophrenia: Systematic review of randomized controlled trials and meta-analysis. General hospital psychiatry. 2020;62:13-20. | 17 studies  (n = 954) | SCZ | Different physical exercise interventions (anaerobic exercise, aerobic exercise, or non-specified exercise) as add-on therapy with a minimum duration of 3 weeks and 8 h of total practice | Non-specific control condition or TAU as usual | The results revealed a significant beneficial effect of physical exercise on negative symptoms. In a subgroup analysis differentiating between aerobic interventions and non-aerobic interventions, aerobic exercise reduced negative and positive symptoms, while this was not the case for non-aerobic interventions. A sensitivity analysis including only studies with a low risk of bias confirmed the effect on negative symptoms, albeit with a small effect size. Moreover, the interventions were heterogeneous, regarding the (often rarely described) types of training, different intensities of practice and different designs of control groups. Based on the characteristics of the studies included, the authors recommend 30 minutes of aerobic exercise at a minimum of 50% VO2 max, for a total session duration of 60 min, with two sessions per week over a minimum of six months. | High quality (++) |
|  |  |  |  |  |  |  |
| Seshadri A, Adaji A, Orth SS, Singh B, Clark MM, Frye MA, et al. Exercise, yoga, and tai chi for treatment of major depressive disorder in outpatient settings: a systematic review and meta-analysis. The primary care companion for CNS disorders. 2020;23(1):24924. | 25 studies (n = 2083) | MD | Exercise (e.g., walking, jogging, and aerobic exercise machines such as the treadmill and stationary bike, strength training), yoga and tai chi. | Any control condition, including other treatment interventions or TAU. | Overall, metanalysis showed a moderate significant clinical effect. However, when only studies (6 studies) with the lowest risk of bias were included, the overall effect size was reduced to low to moderate efficacy. Overall quality of evidence was low. Heterogeneity and publication bias were high. The current meta-analysis of outpatient exercise, yoga, and tai chi for treatment of major depressive disorder suggests that adjunctive exercise and yoga may have small additive clinical effects in comparison to control for reducing depressive symptoms. | High quality (++) |
|  |  |  |  |  |  |  |
| Shannon A, McGuire D, Brown E, O'Donoghue B. A systematic review of the effectiveness of group-based exercise interventions for individuals with first episode psychosis. Psychiatry Research. 2020;293:113402. | 5 studies (n = 363) | SCZ | Group-based exercise interventions | TAU or active control group (such as cognitive training or individual-based exercise) | Whilst group-based exercise interventions did not have a significant impact on physical health, there was a significant effect on schizophrenia symptoms, quality of life, cognition, and functioning. The overall risk of bias was high. More high-quality research is required to understand the best mode of delivery of exercise interventions. | High quality (++) |
|  |  |  |  |  |  |  |
| Shimada T, Ito S, Makabe A, Yamanushi A, Takenaka A, Kawano K, et al. Aerobic exercise and cognitive functioning in schizophrenia: An updated systematic review and meta-analysis. Psychiatry research. 2022;314:114656. | 15 studies (n = 593) | SCZ | Any type of aerobic exercise | TAU, minimal physical activity (including stretching or balance training of similar duration), treatment with no physical exercise, or no treatment | Aerobic exercises resulted in significant improvements in global cognition, attention/vigilance, working memory, and verbal learning. Significant improvements in global cognition were observed with group exercise, exercise supervised by exercise professionals, as well as with ≥ 90 min/week and ≥ 12 weeks duration. | High quality (++) |
|  |  |  |  |  |  |  |
| Sawyer C, McKeon G, Hassan L, Onyweaka H, Martinez Agulleiro L, Guinart D, et al. Digital health behaviour change interventions in severe mental illness: a systematic review. Psychological medicine. 2023;53(15):6965-7005. | 14 studies relevant for this review  (n = 1727) | SMI | Digital health behaviour change interventions targeting physical activity/weight loss/cardio-metabolic health | Not specified | Acceptance and feasibility of digital physical activity interventions were generally high. Peer interaction (especially with peer coaches) appeared to be a helpful component. Five out of nine studies reporting on weight loss and/or physical activity outcomes showed some promising results. The only study focusing on mental health outcomes did not find significant improvements. | Acceptable (+) |
|  |  |  |  |  |  |  |
| Su Y, Pan X, Li H, Zhang G. Effects of mind-body therapies on schizophrenia: A systematic review and network meta-analysis. Schizophrenia research. 2024;264:236-47. | 22 studies (n = 2064) | SCZ | Mind-body therapies (for this review relevant interventions: Yoga, Tai Chi, Baduanjin, Yijinjing) | Treatment as usual and rehabili-tation (including routine exercise) | Mindfulness-based therapies may effectively reduce schizophrenia symptoms, with Yoga showing the greatest effects (Yoga was the best intervention to reduce negative symptoms and the second most effective intervention for reducing positive symptoms [after mindfulness]). | High quality (++) |
|  |  |  |  |  |  |  |
| Swora E, Boberska M, Kulis E, Knoll N, Keller J, Luszczynska A. Physical Activity, Positive and Negative Symptoms of Psychosis, and General Psychopathology among People with Psychotic Disorders: A Meta-Analysis. J Clin Med. 2022;11(10). | 27 studies (n = 1664) | SCZ | Aerobic exercise, yoga, HIIT, psychoeducation (if the focus was mostly on a psychosocial intervention, including physical activity guidelines and examples of exercises, and other exercise interventions (if various types of exercise were combined in one training) | Not specified | Participating in physical activity interventions (or higher levels of physical activity) were associated with lower levels of positive and negative symptoms, and general psychopathology. | High quality (++) |
|  |  |  |  |  |  |  |
| Tavares VDO, Rossell SL, Schuch FB, Herring M, Menezes de Sousa G, Galvão-Coelho NL, et al. Effects of exercise on cognitive functioning in adults with serious mental illness: A meta analytic review. Psychiatry research. 2023;321:115081. | 16 studies  (n = 936) | SMI | Any exercise intervention (mainly aerobic exercise, but also strength training, dance, tai chi, etc.) lasting between 3 and 16 weeks. | TAU, active control (e.g. stretching, sedentary games, art therapy), waitlist | Exercise had a large positive effect on reasoning and problem solving, a small effect on executive function, and no effect on other cognitive domains in people with SMI, according to a meta-analysis based on 15 randomised controlled clinical trials.  Subgroup analysis showed that in people with depression, there was a moderate effect on executive function and a large effect on processing speed. In people with schizophrenia, there was a large effect on reasoning and problem solving. | High quality  (++) |
|  |  |  |  |  |  |  |
| Vogel JS, van der Gaag M, Slofstra C, Knegtering H, Bruins J, Castelein S. The effect of mind-body and aerobic exercise on negative symptoms in schizophrenia: A meta-analysis. Psychiatry research. 2019;279:295-305. | 22 studies (n = 1249) | SCZ | Physical exercise interventions including mind-body exercise (such as yoga or tai chi), aerobic exercise and resistance training | Any control group (active and/or inactive control conditions) | The meta-analysis showed a medium significant effect in favour of any exercise intervention versus any control condition. Mind-body exercise and aerobic exercise respectively showed a medium significant effect and a small significant effect versus any control condition. The effect of resistance training could not be examined due to lacking studies. The overall heterogeneity was high, and the overall methodological quality was poor. | High quality (++) |
|  |  |  |  |  |  |  |
| Wu Y, Yan D, Yang J. Effectiveness of yoga for major depressive disorder: A systematic review and meta-analysis. Frontiers in psychiatry. 2023;14:1138205. | 34 studies (n = 2341) | MD | Yoga | Any randomized control group | Yoga has a wide patient acceptance and can significantly improve depressive symptoms (moderate effect on depressive symptoms found in the meta-analysis) and anxiety (negligible effect found in the meta-analysis) in patients with MD. The quality of evidence was low to moderate. | High quality (++) |
|  |  |  |  |  |  |  |
| Xie Y, Wu Z, Sun L, Zhou L, Wang G, Xiao L, et al. The effects and mechanisms of exercise on the treatment of depression. Frontiers in psychiatry. 2021;12:705559. | 7 studies relevant for this review  (n = 508) | MD | Exercise therapy | Not specified | Exercise had therapeutic effects on depression in all age groups, as a single therapy, an adjuvant therapy, or a combination therapy, and the benefits of exercise therapy are comparable to traditional treatments for depression. Moderate intensity exercise is enough to reduce depressive symptoms, but higher-dose exercise is superior to improve for overall functioning. | Unacceptable (0) |
|  |  |  |  |  |  |  |
| Xu Y, Cai Z, Fang C, Zheng J, Shan J, Yang Y. Impact of aerobic exercise on cognitive function in patients with schizophrenia during daily care: A meta-analysis. Psychiatry research. 2022;312:114560. | 22 studies (n = 1014) | SCZ | Aerobic exercise | Inactive (TAU, waitlist) or active control groups (occupational therapy, table football, cognitive remediation) | Aerobic exercise led by an occupational therapist led to improvements in global cognition and was also associated with improved verbal learning and memory, reasoning and problem-solving. Aerobic exercise also showed a long-term beneficial effect on global cognition in people with schizophrenia. | High quality (++) |
|  |  |  |  |  |  |  |
| Yin J, Sun Y, Zhu Y, Alifujiang H, Wang Y, An S, et al. Effects of yoga on clinical symptoms, quality of life and social functioning in patients with schizophrenia: A systematic review and meta-analysis. Asian J Psychiatr. 2024;93:103959. | 19 studies (n = 1274) | SCZ | Yoga | Active (e.g., physical or aerobic exercise) or passive (e.g., TAU or waitlist) control | Yoga was associated with a reduction in clinical symptoms, primarily in short term, while demonstrating a sustained effect only on negative symptoms over the long-term with improved quality of life and social functioning. A medium effect size was observed in clinical symptoms comparing yoga to passive control group (but no significant effect over the active control group). | High quality (++) |
|  |  |  |  |  |  |  |
| Yip ALK, Karatzias T, Chien WT. Mindfulness-based interventions for non-affective psychosis: a comprehensive systematic review and meta-analysis. Annals of Medicine. 2022;54(1):2339-52. | 12 studies relevant for this review  (n = 783) | SCZ | Mindfulness based interventions such as yoga (but also acceptance-based mindfulness and compassionate meditation) | Active control groups using e.g., psychoeducation or work-related training with or without TAU, or TAU only. | Yoga interventions led to a significantly higher reduction in psychotic symptoms than TAU and/or other psychosocial interventions. In addition, quality of life and clinical functioning were improved significantly. | High quality (++) |
|  |  |  |  |  |  |  |
| Yu Q, Wong KK, Lei OK, Nie J, Shi Q, Zou L, et al. Comparative Effectiveness of Multiple Exercise Interventions in the Treatment of Mental Health Disorders: A Systematic Review and Network Meta-Analysis. Sports Med Open. 2022;8(1):135. | 101 studies relevant for this review (n not reported separately) | SMI | Exercise (aerobic exercise, resistance training, mind body exercise, stretching, multimodal exercise, other types of exercise) | TAU, health education, or other types of exercise not applied in the experimental group | Multimodal exercise had the highest probability of being the most efficacious exercise for relieving depressive symptoms, resistance training for positive symptoms in SCZ and multimodal exercise for negative symptoms in SCZ. Exercise frequency moderated the effectiveness of mind body exercise on negative symptoms in SCZ. The findings should be treated with caution as the quality of evidence was low to moderate. | High quality (++) |
|  |  |  |  |  |  |  |
| Ziebart C, Bobos P, MacDermid JC, Furtado R, Sobczak DJ, Doering M. The efficacy and safety of exercise and physical activity on psychosis: A systematic review and meta-analysis. Frontiers in psychiatry. 2022;13:807140. | 24 studies (n = 1426) | SCZ | Any form of exercise or physical activity (e.g., dancing, running, walking, yoga, strength training, HIIT, and lifestyle modifications such as increased daily walking and mobility) provided within a hospital setting | TAU, other forms of exercise (e.g., stretching), self-directed care, treatment with another healthcare professional | Aerobic had more pronounced effects when compared to TAU in PANSS positive, negative, general, and total scores. Yoga when compared to TAU showed no differences in PANSS subscale and overall scores. The quality of evidence was low to moderate due to a high risk of bias and imprecision. | High quality (++) |

Note: BD: bipolar disorder, MD: major depression, SCZ: schizophrenia, SMI: severe mental illnesses, TAU: treatment as usual

**S6: Included reviews on diet interventions**

| Reference | Included studies / n | Popu-lation | Interventions | Control group | Main results | SIGN checklist |
| --- | --- | --- | --- | --- | --- | --- |
| Altun A, Brown H, Szoeke C, Goodwill AM. The Mediterranean dietary pattern and depression risk: A systematic review. Neurology, Psychiatry and Brain Research. 2019;33:1-10. | 3 studies relevant for this review (n = 253) | MD | Mediterranean diet | Not specified | Individuals with depression who followed a modified Mediterranean diet for a duration of six months suffered from fewer depressive episodes when compared to the control group. | Low quality (-) |
|  |  |  |  |  |  |  |
| Aranburu E, Matias S, Simón E, Larretxi I, Martínez O, Bustamante MÁ, Fernández-Gil MDP, Miranda J. Gluten and FODMAPs Relationship with Mental Disorders: Systematic Review. Nutrients. 2021 May 31;13(6):1894. doi: 10.3390/nu13061894. PMID: 34072914; PMCID: PMC8228761. | 1 study relevant for this review (n = 16) | SMI | Dietary interventions containing low gluten and fermentable oligosaccharides, disaccharides, monosaccharides, and polyols (FODMAP). | Not specified | Limiting or ruling out gluten might be beneficial to improve cognition in schizophrenia. | Low quality (-) |
|  |  |  |  |  |  |  |
| Murta L, Seixas D, Harada L, Damiano RF, Zanetti M. Intermittent Fasting as a Potential Therapeutic Instrument for Major Depression Disorder: A Systematic Review of Clinical and Preclinical Studies. International journal of molecular sciences. 2023;24(21). | 1 study relevant for this review (n = 100) | MD | Intermittent Fasting (religious fasting) | Not specified | No difference was found in depressive symptoms between the intervention group (Ramadan fasting) and the control group (no fasting), but there were reductions in weight, BMI and body fat. | Low quality (-) |
|  |  |  |  |  |  |  |
| Pearsall R, Praveen KT, Pelosi A, Geddes J. Dietary advice for people with schizophrenia. Cochrane Database of Systematic Reviews. 2016(3). | 0 studies identified | SCZ |  | Standard care |  | High quality (++) |
|  |  |  |  |  |  |  |
| Rocks T, Teasdale SB, Fehily C, Young C, Howland G, Kelly B, et al. Effectiveness of nutrition and dietary interventions for people with serious mental illness: systematic review and meta-analysis. Med J Aust. 2022;217 Suppl 7(Suppl 7):S7-s21. | 25 studies (n = 2863) | SMI | Nutrition related interventions | TAU | There is only limited evidence for the effectiveness of nutrition interventions in improving metabolic syndrome risk factors in people with SMI. Subgroup analyses suggest higher effectiveness when interventions are delivered in individual sessions and/or by dietitians. | High quality (++) |
|  |  |  |  |  |  |  |
| Swainson J, Reeson M, Malik U, Stefanuk I, Cummins M, Sivapalan S. Diet and depression: A systematic review of whole dietary interventions as treatment in patients with depression. J Affect Disord. 2023;327:270-8 | 5 studies (n = 361) | MD | Dietary interventions (comprehensive diet plan or way of eating, e.g., caloric restriction, Mediterranean diet) | Not specified | The findings provide preliminary evidence for the positive impact of dietary interventions in people with depression. While all studies reported some form of mood improvement in the intervention (often a form of caloric/carbohydrate restriction) compared to the control group, the heterogeneity among studies prevented an adequate comparison and hinders the generalizability of findings. | Acceptable (+) |
|  |  |  |  |  |  |  |
| Teasdale SB, Ward PB, Rosenbaum S, Samaras K, Stubbs B. Solving a weighty problem: systematic review and meta-analysis of nutrition interventions in severe mental illness. The British Journal of Psychiatry. 2017;210(2):110-8. | 26 studies (n = 1630) | SMI | Studies on dietary patterns: cooking classes, budgeting, and grocery shopping; additional exercise, smoking cessation, and psychosocial interventions (e. g. CBT or motivational interviewing) | TAU with or without written physical health information | Interventions led to significant weight loss, reduced body mass index, decreased waist circumference, and lower blood glucose levels. Larger effect sizes were found for dietitian-led interventions and studies delivered at antipsychotic initiation. | Unacceptable (0) |
|  |  |  |  |  |  |  |
| Tully A, Smyth S, Conway Y, Geddes J, Devane D, Kelly JP, et al. Interventions for the management of obesity in people with bipolar disorder. Cochrane Database of Systematic Reviews. 2020(7). | 0 studies identified | BD | Among others: non-pharmacological interventions comprising dietary, exercise, behavioural, and multi-component interventions | Intervention versus inactive comparator | There is a need for more randomized, controlled, and blinded intervention studies powered to detect changes in mental health rather than metabolic outcomes. | High quality (++) |

Note: BD: bipolar disorder, MD: major depression, SCZ: schizophrenia, SMI: severe mental illnesses, TAU: treatment as usual

**S7: Included reviews on sleep interventions**

| Reference | Included studies / n | Popu-lation | Interventions | Control group | Main results | SIGN checklist |
| --- | --- | --- | --- | --- | --- | --- |
| Bisdounis L, Saunders KEA, Farley HJ, Lee CK, McGowan NM, Espie CA, et al. Psychological and behavioural interventions in bipolar disorder that target sleep and circadian rhythms: A systematic review of randomised controlled trials. Neuroscience and biobehavioral reviews. 2022;132:378-90. | 10 studies relevant for this review (n = 797) | BD | Interpersonal and social rhythm therapy,  CBTi (CBT for insomnia),  Total sleep deprivation,  Combination treatments | All kinds of control group | Meta-analysis for the effect of bright light therapy on depression revealing a medium-to-large post-treatment effect. Small number of studies for each intervention; lack of consistency in protocols and outcomes | High quality (++) |
|  |  |  |  |  |  |  |
| Waite F, Sheaves B, Isham L, Reeve S, Freeman D. Sleep and schizophrenia: From epiphenomenon to treatable causal target. Schizophrenia Research. 2020;221:44-56. | 6 studies relevant for this review (n = 263) | SCZ | CBTi,  Sleep treatment at acute crisis (STAC),  Brief CBT for nightmares | TAU (one study without control group) | Large effect size improvements in sleep; modest improvements in psychotic experiences | Unacceptable (0) |

Note: BD: bipolar disorder, SCZ: schizophrenia, TAU: treatment as usual
